# Helminthic larval stage induces cellular apoptosis via caspase 9-mediated mitochondrial dysfunction

**DOI:** 10.3389/fimmu.2025.1603385

**Published:** 2025-09-25

**Authors:** Leonardo Elias Sternkopf, Ulrich Fabien Prodjinotho, Vitka Gres, Nikolaus Repgen, Katja Steiger, Julia Schluckebier, Chummy S. Sikasunge, Dominik Stelzle, Charles Makasi, Andrea Sylvia Winkler, Bernard J. Ngowi, Nelly Villalobos, Friederike Ebner, Georg Häcker, Philipp Henneke, Clarissa Prazeres da Costa

**Affiliations:** ^1^ Institute for Medical Microbiology, Immunology and Hygiene, TUM School of Medicine and Health, Technical University of Munich (TUM), Munich, Germany; ^2^ Center for Global Health, TUM School of Medicine, Technical University of Munich (TUM), Munich, Germany; ^3^ Institute for Infection Prevention and Control and Center for Chronic Immunodeficiency (CCI), Medical Center and Faculty of Medicine, University of Freiburg, Freiburg, Germany; ^4^ Infection Pathogenesis, School of Life Sciences, Technical University of Munich (TUM), Freising, Germany; ^5^ Institute of Pathology, Technical University of Munich (TUM), Munich, Germany; ^6^ Department of Paraclinical Studies, School of Veterinary Medicine, University of Zambia, Lusaka, Zambia; ^7^ Department of Neurology, Kilimanjaro Christian Medical University College, Moshi, Tanzania; ^8^ National Institute for Medical Research, Muhimbili Research Centre, Dar es Salaam, Tanzania; ^9^ Department of Neurology, TUM School of Medicine, Technical University of Munich (TUM), Munich, Germany; ^10^ Department of Community Medicine and Global Health, Institute of Health and Society, Faculty of Medicine, University of Oslo, Oslo, Norway; ^11^ Mbeya College of Health and Allied Sciences, University of Dar es Salaam, Mbeya, Tanzania; ^12^ Departamento de Patología, Facultad de Medicina Veterinaria y Zootecnia, Universidad Nacional Autónoma de México (UNAM), Mexico City, Mexico; ^13^ Institute of Medical Microbiology and Hygiene, Medical Center - University of Freiburg, Faculty of Medicine, University of Freiburg, Freiburg, Germany; ^14^ Centre for Integrative Biological Signalling Studies, University of Freiburg, Freiburg, Germany; ^15^ German Center for Infection and Research (DZIF), Munich, Germany

**Keywords:** T. solium cyst vesicular fluid, neurocysticercosis, inflammation, apoptosis pathways, caspase 9 activity

## Abstract

**Introduction:**

In human neurocysticercosis (NCC), the cellular and molecular mechanisms of host-parasite interactions triggering brain inflammation and epileptic seizures in Sub-Saharan Africa are poorly understood. Emerging evidence indicates that the viability of the cyst of the pork tapeworm *Taenia solium* determines brain inflammation and, thus, symptom development and disease severity. We have previously shown that while viable cyst-released molecules promote immune regulation and often asymptomatic disease, the fluid from degenerating cysts causes inflammation in microglia and peripheral immune cells, potentially driving immune-mediated pathology. This study aims to elucidate the apoptotic signaling pathways underlying this process and their relevance for symptomatic disease in NCC patients.

**Materials and methods:**

Human and porcine peripheral immune cells, as well as murine microglia, were exposed to *T. solium* cyst vesicular fluid (CVF). Apoptosis signaling pathways were analysed using flow cytometric FLICA (fluorochrome-labeled inhibitors of caspases) caspase 8 and 9 assays, while mitochondrial dysfunction was assessed via TMRE and MitoTracker Deep Red and Green fluorescent probes. Apoptosis-inducing CVF molecules were identified by differential mass spectrometry and functionally tested using specific inhibitors. Caspase activity and soluble mediators (FasL, ROS, TNFα) were measured in NCC asymptomatic and symptomatic patients’ sera, and inflammatory T cell infiltrates expressing caspases near viable and degenerating cysts in naturally infected pig brain slices were examined via immunohistology.

**Results:**

We found that vesicular fluid derived from cysts primarily induced apoptosis and caspase 3 and 9 activity, and only minimal necrosis, in a dose-dependent manner across central and peripheral immune cells. This effect was prominent in CD16^+^ monocytes, microglia, and in CD3^+^ T cell-expressing caspase 3 near degenerating brain cysts. Apoptotic signaling was predominantly mediated by a dynamic remodeling of caspase 9 pathway, accompanied by a significant loss of mitochondrial potential and a sharp decrease in Bid and Bcl2 transcription, favoring the intrinsic over the FasL-dependent extrinsic pathway and mechanisms. This process is primarily mediated by small molecules (< 30 kDa), and remained unaffected by heat and proteinase treatment. Notably, symptomatic NCC patients exhibited elevated FasL levels correlating with increased caspase activity, underscoring the potential contribution of apoptosis to disease pathogenesis.

**Conclusions:**

This study identifies caspase 9-mediated apoptosis as a mechanism of helminth-induced brain inflammation and implicates FasL in symptomatic disease progression. These insights enhance our understanding of NCC immunopathogenesis and may inform future therapeutic strategies targeting apoptotic pathways.

## Introduction

Neurocysticercosis (NCC), a human parasitic disease caused by larval cyst stages of the pork tapeworm *Taenia solium*, remains a major public health concern in endemic regions, particularly in low- and middle-income countries (LMICs) of Sub-Saharan Africa, Latin America, and Southeast Asia (1). NCC is the leading cause of acquired epilepsy worldwide, disproportionately affecting rural populations with limited access to healthcare (2). The pathogenesis of NCC is closely linked to the viability and degenerative state of *T. solium* cysts in the central nervous system (CNS) (3, 4). While viable cysts typically sustain a state of immune tolerance via regulatory T cells (Tregs), leading mainly to asymptomatic or mild disease, cyst degeneration and death are believed to trigger a pronounced inflammatory response, often culminating in symptomatic NCC. This is characterized by epileptic seizures, chronic headaches, and neuroinflammation (4–7). However, the precise cellular and molecular mechanisms by which degenerating cysts modulate immune responses and contribute to neuroinflammation remain incompletely understood. In this study, we explored the implication of apoptosis pathways in regulating these processes.

The host immune response to NCC involves a complex interplay between resident brain cells, including microglia and astrocytes, and activated and infiltrating peripheral immune cells such as monocytes and their macrophage progeny, and T cells (3, 8–10). Recent studies indicate that microglia, the most frequent mononuclear cells of the resting CNS (11), play a central role in recognizing and responding to degenerating cyst materials by secreting pro-inflammatory mediators (TNFα, IFNγ, IL-1ß, IL-18, IL-12) (8, 10, 12) and phagocytosing damaged neurons and neurotoxic aggregates. Thus, they potentially shape the neuroimmune microenvironment and preserve nervous tissue homeostasis (3, 12, 13). Additionally, systemic immune dysregulation, including monocyte and lymphocyte recruitment, activation, and apoptosis, has been observed in NCC (5, 14–19). This suggests that parasite-derived factors may influence immune cell survival and function. While previous work has highlighted the main role of cytokine storms, cyst-associated mechanical and synaptic obstruction in NCC-related neuroinflammation, the contribution of apoptotic pathways, particularly caspase-dependent signaling, to disease progression has not been fully clarified.

Apoptosis, the best understood form of regulated cell death, is essential for many important biological processes, such as in embryonic development, maintenance of cellular homeostasis, and negative selection of T lymphocytes (20–22). Thus, it is a fundamental mechanism governing immune homeostasis and pathogen clearance. It is orchestrated by two major pathways, both of which are found in parasitic helminth infections: the extrinsic and the intrinsic pathways. The extrinsic pathway is mediated by the activation of death receptors such as Fas and TNF receptor superfamily members. The intrinsic mitochondrial pathway, on the other hand, is induced through cellular stressors, like hypoxia or heat shock, and is tightly regulated by the balance of pro- and anti-apoptotic Bcl-2 family proteins (20, 23–25). The intrinsic pathway is particularly critical for CNS pathology, as mitochondrial dysfunction, caspase 9 activation, and increased systemic immune cell apoptosis have been implicated in neurodegenerative diseases and inflammatory responses of the CNS (26, 27). Thus, it seems imperative to thoroughly characterize cyst-regulated central and peripheral cell apoptosis and dissect their molecular signatures, particularly in relation to caspase-driven mitochondrial dysfunction.

Our previous research demonstrated that degenerating cysts promote microglial and macrophage activation and apoptosis, yet the effect on peripheral immune cells and the precise molecular triggers remained unidentified. Here, we sought to define the apoptotic signaling pathways involved in NCC pathogenesis, focusing on the role of caspase-mediated mitochondrial dysfunction. Using a combination of *in vitro* cellular assays, caspase activity profiling, mass spectrometry-based proteomics, and animal infection and patient serum analyses, we characterized the differential immune responses and apoptosis elicited in the presence of viable versus degenerating cysts. We further examined apoptotic gene (e.g. Bid, Bcl2) regulation by cyst molecules and provided insights into the relative contributions of intrinsic versus extrinsic apoptotic pathways during NCC pathogenesis through caspase 3, 8, 9 activity, and association with FasL levels in symptomatic and asymptomatic NCC patients.

## Materials and methods

### Animals and *T. solium* cyst products

C57BL/6 mice were purchased from Envigo (Germany) or bred in-house and maintained under specific pathogen-free conditions at the Institute of Medical Microbiology, Immunology, and Hygiene (MIH), Animal facility, TU Munich. All experiments were performed in accordance with and approved by local government authorities of Regierung von Oberbayern (Az. 55.2-1-54-2532-145-17). Cyst vesicular fluid (CVF) was prepared as described previously (3). *T. solium* cysts were collected from the muscle tissue of a heavily infected pig at the University of Zambia School of Veterinary Medicine (ethical approval Ref. 2018-Mar-002/0005948). Following collection, the cysts were washed with phosphate-buffered saline (PBS) (Merck, Cat. No. D8662). For CVF preparation, the cysts were transferred to a sterile dish, cut open with a scalpel, and the released fluid was collected. The collected fluid was sterile filtered through 0.45 µm size filters, centrifuged at 15.000 g for 60 min at 4 °C. Then, materials were immediately treated with Phenylmethylsulphonyl fluoride (PMSF, final concentration 5 mM) and leupeptin (final concentration 2.5 µM), and aliquoted at a concentration of 1 mg/ml in PBS. All prepared materials were tested for endotoxin (levels < 0.05 EU/ml) and stored at -80 °C until use.

### Human PBMC and plasma from patients with NCC and controls

Human plasma from healthy controls and persons with NCC was examined for soluble apoptosis-inducing mediators. The patients were recruited within the TOPANA study, a prospective cohort study implemented in rural Tanzania. The study received ethical approval by the Klinikum Rechts der Isar (Technical University of Munich, Germany) Ethical Committee (Reference 33/19S) and by the National Ethics Health Research Committee (NatREC) of Tanzania (Reference NIMR/HQ/R.8c/Vol.I/1808 for TOPANA and NIMR/HQ/R.8a/Vol.IX/2597 for SOLID). The protocol and study design of the study have been published elsewhere (28, 29). In brief, patients were recruited at three district hospitals (Tosamaganga, Chunya, Vwawa). NCC diagnosis was done in July 2021 according to the latest Del Brutto criteria, using computer tomography (CT) scan and laboratory testing (LDBio cysticercosis Western Blot IgG, apDia Cysticercosis Ag-ELISA) (30, 31). Patients with NCC were classified as symptomatic through the presence or history of severe progressive headaches and/or epileptic seizures and having parenchymal and extraparenchymal NCC with active lesions and mostly degenerating/calcified cysts (28). Asymptomatic NCC patients had no history of epileptic seizures and/or severe progressive headaches but had a mix of viable and degenerating cysts, with most being viable. Participants were tested for HIV by serology, *Mycobacterium tuberculosis* in the sputum, *Strongyloides* spp. in the feces, and underwent fundoscopy for the exclusion of ocular NCC according to the IDSA/ASTMH recommendations (32). Blood samples were taken from the study participants and after giving informed consent. Peripheral blood mononuclear cells (PBMC) were isolated as previously described (33). Plasma was separated from the cellular components of the blood using density gradient centrifugation (700 x g, 25 min, 20 °C), and stored at -80 °C until usage. The cohort included NCC non-infected Tanzanian individuals as controls (group 1), NCC asymptomatic individuals (group 2) and symptomatic NCC patients with epilepsy and/or severe progressive headaches (group 3). The participants included in this study were age and sex-matched (see detail in **Table 1**).

**Table 1:** Study participant groups.

Parameter	Controls	NCC asymptomatic	NCC symptomatic
Total number	6	6	6
Sex (male/female)	3/3	3/3	3/3
Age (range(median))	28 – 60 (40)	27 – 61 (43)	24 – 56 (38)
Symptoms	No	No	Epileptic seizures and/or severe progressive headaches

### Human cell isolation from healthy volunteers, stimulation, and analysis

The study was approved by the local ethical committee of the Technical University of Munich (Reference: 215/18S), and all individuals included in the study consented to enrollment. Human PBMC were isolated from whole blood samples of healthy volunteers from mixed genders using density gradient centrifugation and stored in liquid nitrogen as described in (33). Human unlabeled monocytes were purified from freshly isolated human PBMCs using the Miltenyi Pan Monocyte Isolation Kit, human (Miltenyi, Germany, Cat. No. 130-096-537), according to the manufacturer’s protocol. The purity of monocytes was routinely ≥ 80%. 2.5x10^5^ cells per well were plated in 96 U-bottom well plates and stimulated with CVF (0.01 – 5 µg/ml) and incubated for 0–72 h at 37 °C, 5% CO_2_. Staurosporine (TOCRIS, Bio-techne, Cat. No. 1285) (100 nM or 500 nM) was used to induce apoptosis. After incubation period, surface markers (CD3, CD4, CD8, CD14, CD16 (all antibodies from Biolegend: Cat. No. 317331, 317433, 344723, 325607, 302025), were analyzed by flow cytometry on a CytoFLEX S (Beckman Coulter). Early apoptotic (Annexin V^+^/PI^-^, late apoptotic (Annexin V^+^/PI^+^), and necrotic (Annexin V^-^/PI^+^) cell populations were determined by Annexin V/PI kit (Biolegend, Cat. No. 640914). For intracellular staining, cells were fixed and permeabilized with BD Cytofix/Cytoperm (BD Biosciences, Cat. No. 554714) for detection of active caspase 3 (BD Pharmingen, Cat. No. 570332). FAM FLICA™ Caspase-8 and Caspase-9 Kits (Bio-Rad, Cat. No. ICT099, ICT912) were used for detection of active caspases 8 and 9.

### Column fractionation and CVF-fragment induction of apoptosis

To determine the role of proteins in the CVF in the induction of apoptosis on PBMCs, active proteins in the CVF were inactivated by heat-inactivation using 56 °C for 1h, 56 °C for 3h, 99 °C for 1h, and 99 °C for 3h and 10µg/ml Proteinase K (Sigma-Aldrich, Cat. No. P2308) digestion overnight at 37 °C, 5% CO_2_. SDS page and Coomassie staining were performed to confirm effective protein inactivation. CVF was additionally treated with the cysteine protease inhibitor E-64 (Sigma-Aldrich, Cat. No. E3132) 10µM for 1 hour to inhibit cysteine proteases (e.g. Cathepsins). After protein inactivation and protease inhibition, PBMCs were stimulated with 2.5 µg/ml pretreated CVF for 72 h, and apoptosis induction was evaluated using Annexin V/PI. Furthermore, CVF was separated according to molecular size using 30 kDa column fractionation (GE Healthcare, Cat. No. 17VS50039A). Flow through and retarded CVF fractions (< 30 kDa, > 30 kDa) were used for stimulation of PBMCs and apoptosis was detected.

### Mitochondrial function and membrane potential evaluation

To measure mitochondrial damage, PBMCs were stained with Tetramethylrhodamine, ethyl ester, perchlorate (TMRE) (ThermoFisher Scientific, Cat. No. T669) and MitoTracker following CVF stimulation. For TMRE staining, TMRE was diluted to 10nM in RPMI (Merck, Cat. No. R0883) (10% FCS (Merck, Cat. No. S0615) and 1% Penicillin/Streptomycin (Merck, Cat. No. P4333) and cyclosporin H (Enzo, Cat. No. 83602-39-5) (final concentration 0.5 µM), and incubated with CVF-treated PBMCs for 30 minutes at 37 °C to assess the loss of mitochondrial transmembrane potential. For MitoTracker staining, PBMC were incubated with MitoTracker Deep Red FM (ThermoFisher Scientific, Cat. No. M46753) and MitoTracker Green FM (ThermoFisher Scientific, Cat. No. M7514) in PBS for 60 minutes at 37 °C to evaluate the mitochondrial potential and mass loss, respectively (34).

### Porcine cell isolation, stimulation, and analysis

Porcine blood samples were obtained from freshly slaughtered pigs at a local slaughterhouse. The blood was collected in 500 ml bottles containing 1g EDTA (Ethylenediaminetetraacetic acid) anticoagulant. Samples were stored at 4 °C within an hour of collection and processed within 24 hours.

Porcine PBMCs were isolated using SepMate™ isolation tubes (Stemcell Technologies, Cat. No. 85460) and Histopaque^®^-1077 (Merck, Cat. No. 10771). The blood was first diluted 1:1 with sterile PBS/2% fetal calf serum (FCS) (Merck, Cat. No. S0615), and then layered onto Histopaque within centrifugation tubes. PBMCs were separated using a density gradient. Centrifugation was performed at 700xg for 25 min with the lowest acceleration and break. The PBMC layer was collected using a single-use pipette. PBCMs were washed twice with PBS + 2% FCS. Remaining erythrocytes were lysed using 0.5 ml erythrocyte lysis buffer (0.01 M KHCO_3_, 0.155 M NH_4_Cl, 0.1 mM EDTA) per 10 ml blood for 4 min at RT.

PBMCs were resuspended in RPMI (Merck, Cat. No. R8758) supplemented with 10% FCS (Merck, Cat. No. S0615), 100 U/ml penicillin, 100 µg/ml streptomycin (Merck, Cat. No. P4333). Porcine monocytes were separated from freshly isolated PBMCs using the autoMACS separator (Miltenyi Biotec). PBMCs from each animal were rebuffered in autoMACS running buffer (Miltenyi Biotec, Cat. No. 130-091-221). Cells were labeled with CD14 MicroBeads (Miltenyi Biotec, clone Tük4, Cat. No. 130-050-201) and separated according to the manufacturer’s instructions. CD14^+^ monocytes were pelleted and resuspended in RPMI. Trypan blue staining and automated cell counting were used to determine the number of viable monocytes and PBMCs.

2.5–5 x 10^5^ cells of porcine PBMCs and 3 x10^5^ cells of porcine monocytes were plated per well and stimulated with 2.5 µg/ml CVF for 0–72 h at 37 °C and 5% CO_2_. After incubation, porcine-specific anti-CD4α (clone 74-12-4, mouse IgG2b, inhouse + anti-mouse IgG2b, clone R12-3, BD Biosciences, Cat. No. 743177), anti-CD8α (clone 11/295/33, mouse IgG2a, inhouse + anti-mouse IgG2a, clone RMG2 A-62, BioLegend, Cat. No. 407117), anti-CD3e (clone BB-23-8E6-8C8, BD Biosciences, Cat. No. 743177), anti-CD172a (clone 74-22-15, mouse IgG1, inhouse + anti-mouse IgG1, clone X-56, Miltenyi, Cat. No. 130-096-593) and CD14 (clone Tük4, Bio-Rad, Cat. No. MCA1568P647) antibodies were used to characterize cell populations on an Attune NxT Flow cytometer (Thermo Fisher Scientific). Induction of apoptosis was detected with Annexin V/PI kit (Invitrogen, Cat. No. 13242) and Staurosporine (500 nM) (Sigma-Aldrich, Cat. No. S6942) served as a positive control.

### Immunohistology of naturally infected pig brain slices

Ten 2 - 4-year-old pigs (6 females and 4 males, 4 uninfected and 6 naturally infected with *T. solium* cysticercosis, were purchased from rural rearers. The animals were diagnosed for cysticercosis by tongue examination. Pigs were humanely euthanized at the Faculty of Veterinary Medicine and Zootechnics in accordance with Mexican official standard NOM-033-SAG/ZOO-2014. Blood samples were collected immediately after euthanasia, and brain tissue was dissected. Brain samples were fixed in neutral buffered 10% formalin for 48 hours. After dehydration, brain tissue was embedded in paraffin blocks.

Serial sections of 2µm slices were mounted on Superfrost (HE) or Superfrost Plus (IHC) slides using a rotary microtome (ThermoFisher Scientific, Cat. No. HM355S). The sections were stained with Hematoxylin-Eosin to evaluate general pathological changes using standard protocols. In brief, the wax was removed with xylene and then passed through different changes of alcohol to remove the xylene. After rehydration with distilled water, the sections were stained with hematoxylin for 8 minutes. Following rinsing with tab water, counterstaining with 1% alcoholic eosin was performed for 4 minutes. Slides were dehydrated by passing through different changes of alcohol and cleared with xylene.

To visualize and localize cell populations in the granulomatous regions surrounding *T. solium* cysts in the pig brains, immunohistochemical (IHC) staining was performed using an autostainer (Bond RXm system, Leica). In brief, the sections were deparaffinized with the Leica Kit de-wax (Leica, Cat. No. AR9222) and rehydrated by washing with decreasing alcohol concentrations. Epitope retrieval was done with heat induction using citrate buffer (pH=6) for 20 minutes (40 minutes for anti-CD79a). Endogenous peroxidase activity was blocked by incubating the sections with 3% hydrogen peroxidase (Leica) for 5 minutes, and non-specific binding sites were blocked with normal goat serum (Abcam, Cat. No. ab138478). Primary antibodies were then added to the sections for 15 minutes at room temperature (anti-CD3 (Cellmarque, Cat. No. MRQ39), anti-CD79a (ThermoFisher, Cat. No. HM57), anti-Iba1 (poly, Wako, Cat. No. 019-19741), and anti-Caspase 3 (Cell Signaling, Cat. No. 5A1E)). Antibody detection was done with a polymer refine detection kit (Leica, Cat. No. DS9800) and Diaminobenzidine (DAB) (Merck, Cat. No. D12384) was used for visualization. The sections were counterstained with hematoxylin for 10 minutes and dehydrated with increasing alcohol concentrations and xylene. Pertex mounting medium (Histolab, Cat. No. SEA-0100-00A) was used to mount the sections. All antibodies were tested for cross-reactivity in pigs and adequate positive controls were used in each staining run.

### Analysis of histological pig brain slides

Histology was evaluated under the supervision of a board-certified veterinary Pathologist (K.S.). For digital image analysis, the software QuPath [v0.5.1] was used. To quantify CD3^+^ and CD79a^+^ cells, 8–10 rectangles (1,25 mm x 0,875 mm) covering the area around the cysts were drawn for automated cell counting, and positive and negative cells were counted automatically with QuPath software.

### Mouse microglia isolation, culture, and stimulation

Newborn mice (P1-P4) were decapitated, and the olfactory bulbs and the cerebellum were removed, as well as the meninges and choroid plexus. Tissue was dissociated and filtered through 100 µl cell strainer. Cells were centrifuged for 5 min at 1500 RPM at 4 °C. The cell pellet was resuspended in 5 ml of DMEM (Dulbecco’s Modified Eagle Medium) high glucose (Gibco, Cat. No. 11965092) (10% FCS, 0.5% Ciprofloxacin) and plated on a T25 culture flask, which was previously coated overnight with 5 ug/ml Poly-L-Lysine (Merck, Cat. No. P8920). The T25 culture flasks were incubated for 12 days with repeated medium exchange at 37 °C in a 5% CO_2_ humidified incubator. 48h before further analysis, 20ng/ml of M-CSF (Macrophage colony-stimulating factor) (Pepro-Tech, Cat. No. 315-02) was added to the cultures. The cultures were trypsinized in 0.05% Trypsin-EDTA for 15 minutes at 37 °C, washed with medium, scraped, and seeded to 96-well flat-bottom plate (Thermo Fisher Scientific), with a density of 1 x 10^5^ cells per well. After resting overnight, cells were stimulated with CVF (2.5 µg/ml) for 0 - 72h, and, either lysed for qPCR (quantitative polymerase chain reaction), or proceeded for active caspases detection with intracellular staining for cleaved caspase 3 (Bio-techne, R&D Systems, Cat. No. IC835G) and with FLICA caspase assays for active caspase 8 (Bio-Rad, Cat. No. ICT099) and 9 (Bio-Rad, Cat. No. ICT960) using flow cytometry.

### ELISA

Commercially available ELISA kits were used according to the manufacturer’s instructions. Human TNFα ELISA (R&D Systems, Cat. No. DTA00D) and FasL (R&D Systems, Cat. No. DFL00B) were used to evaluate the levels of both apoptosis mediators in the culture supernatants of CVF-stimulated PBMCs from healthy individuals and in plasma samples from NCC patients and controls from Tanzania.

### Intracellular ROS detection

An intracellular fluorometric reactive oxygen species (ROS) assay (Cell Biolabs, Cat. No. STA-342) was used according to the manufacturer’s instructions to measure the intracellular ROS activity within the CVF-stimulated PBMCs. Briefly, human PBMCs were stimulated with CVF 2.5 µg/ml. After 72 h, cells were washed twice with PBS, and 100 µl of 1X cell-permeable fluorogenic probe Dichlorodihydrofluorescin diacetate (DCFH-DA) were added to the wells and incubated at 37 °C, 5% CO_2_. After 45 min, cells were washed twice with PBS, and the fluorescence intensity, proportional to intracellular ROS activity to the DCF, was measured with a CLARIOstar Plus plate reader (BMG LABTECH) at 480 nm/530 nm. 100 µM and 1000 µM H_2_O_2_ were added to the cells as positive controls.

### RNA extraction and qPCR

For microglia RNA extraction, cells were treated with RLysis buffer containing 1% β-mercaptoethanol, and RNA was extracted using the Extractme Total RNA Micro Spin kit (BLIRT, Cat. No. EM31.1-050) according to manufacturer’s information. RNA was reversely transcribed to cDNA with the SuperScript™ IV VILO mix (ThermoFisher Scientific). qRT-PCR was performed with ABsolute qPCR SYBR Green (Thermo Fisher Scientific) at the LightCycler 480 (Roche). ΔΔCt was used to calculate relative gene expression and differences. The oligonucleotide sequences used for the qRT-PCR are detailed in **Table 2**.

**Table 2:** The oligonucleotide sequences used for the qRT-PCR.

Gene	Forward primer (5’-3’)	Reverse primer (5’-3’)
Gapdh	ACTCCACTCACGGCAAATTC	TCTCCATGGTGGTGAAGACA
TNFα	TCGTAGCAAACCACCAAGTG	CCTTGTCCCTTGAAGAGAACC
Caspase 3	CTCGCTCTGGTACGGATGTG	TCCCATAAATGACCCCTTCATCA
Caspase 8	TGCTTGGACTACATCCCACAC	GTTGCAGTCTAGGAAGTTGACC
Caspase 9	GGCTGTTAAACCCCTAGACCA	TGACGGGTCCAGCTTCACTA
FasL	TCCGTGAGTTCACCAACCAAA	GGGGGTTCCCTGTTAAATGGG
Bid	GCCGAGCACATCACAGACC	TGGCAATGTTGTGGATGATTTCT
Bcl-2	GCTACCGTCGTGACTTCGC	CCCCACCGAACTCAAAGAAGG

### Mass spectrometry analysis of CVF

The mass spectrometry analysis of *T. solium* CVF was performed as previously described (3). The retrieved data were analyzed using the WormBase Parasite database for *T. solium* (Version: WS282, October 2022) to identify apoptosis-related molecules in the CVF.

### Statistical analysis

For statistical analysis, the Prism software (Version 10.1.2) (GraphPad Software, LLC, San Diego, CA, USA) was used. For direct comparisons between two groups, a Mann-Whitney test was employed. For more than two groups, we applied a Kruskal-Wallis test followed by Dunn’s multiple comparison test and a two-way ANOVA test followed by Tukey’s multiple comparison test if more time points were observed. *P* values < 0.05 were considered statistically significant.

## Results

### The vesicular fluid from degenerated *T. solium* cyst (CVF) causes apoptosis but not necrosis in human T cells and monocytes

**Figure 1:**
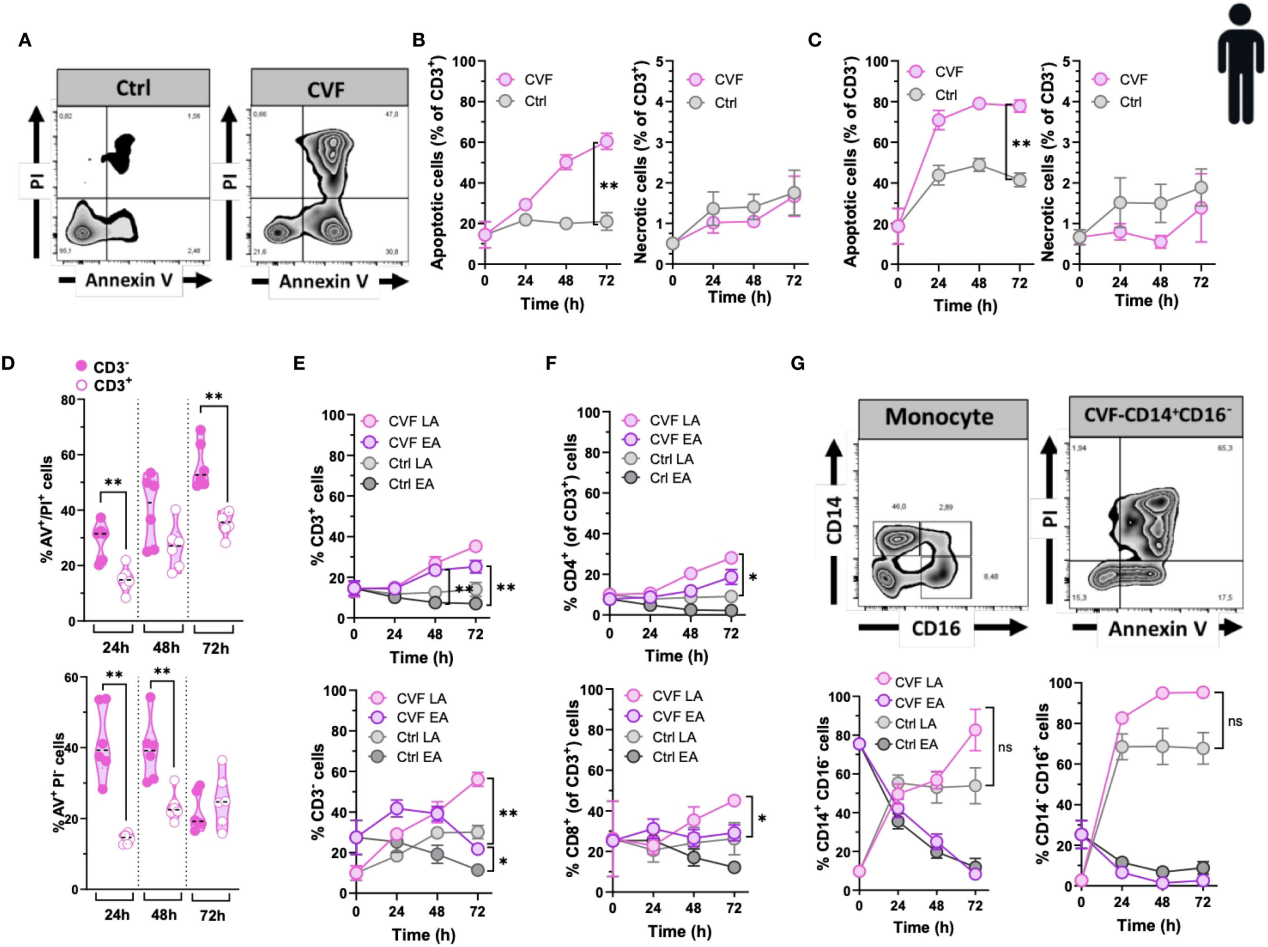
The vesicular fluid of *T. solium* cyst (CVF) causes apoptosis but not necrosis in human T cells and monocytes. **(A)** FACS plots of 72h CVF-stimulated (right panel) and non-stimulated (control, left panel) PBMCs for detecting early (Annexin V^+^/PI^-^) and late (Annexin V^+^/PI^+^) apoptotic and necrotic (Annexin V^-^/PI^+^) cell populations using Annexin V/PI-gating. **(B, C)** CVF-induced apoptosis and necrosis in CD3^+^ **(B)** and CD3^-^ **(C)** cell populations. **(D)** A direct comparison between CD3^-^ and CD3^+^ cells for late (upper panel) and early (lower panel) apoptosis induced by CVF as compared to control. **(E, F)** Time-dependent induction of early and late apoptosis in CD3^+^ **(E)** (upper panel) and CD3^-^ **(E)** (lower panel), and in CD4^+^ **(F)** (upper panel) and CD8^+^ **(F)** (lower panel) cell populations. **(G)** CVF-induced apoptosis in human monocytes in a time-dependent manner. **(G)** (upper panels) FACS plots of the characterization of monocyte subpopulations (upper left) and apoptosis in CD14^+^CD16^-^ monocytes (upper right). **(G)** (lower panels) Early and late apoptosis induced by CVF in classical (CD14^+^CD16^-^) (lower left) and non-classical (CD14^-^CD16^+^) (lower right) monocyte subpopulations after 72h culture. Data information: Graphs show data from 6–8 different samples. Statistical analysis was performed using a Two-way ANOVA followed by Tukey’s multiple comparison tests **(B, C, E–G)** and a Mann-Whitney test **(D)**. Data are represented as means ± SEM. *P < 0.05; **P < 0.01.

Our previous findings indicated that cyst-derived vesicular fluid (CVF) from *T. solium* cysts promotes apoptosis in alveolar macrophages (3). Here, we extended our investigation to assess the pro-apoptotic effects of CVF on various peripheral immune cell populations. To evaluate the impact of CVF on PBMCs, we analyzed both early apoptotic (Annexin V^+^/PI^-^) and late apoptotic (Annexin V^+^/PI^+^) cell populations by Annexin V/PI staining. Our data revealed that CVF induced significant apoptosis in PBMCs (**Figure 1A**; **Supplementary Figure S1**). This effect was concentration-dependent and predominantly restricted to apoptosis rather than necrosis, affecting both CD3^+^ T cells (**Figure 1B**) and CD3^-^ cells (non-T cells) (**Figure 1C**) in a dose-dependent manner (**Supplementary Figure S2**). Notably, CD3^-^ immune cells exhibited a more pronounced apoptotic response compared to CD3^+^ T cells (**Figure 1D**). Furthermore, the apoptotic effect intensified over time, particularly in the late apoptotic CD3^+^ and CD3^-^ cell populations (**Figure 1E**) as well as in CD3^+^CD4^+^ and CD3^+^CD8^+^ T cell subsets (**Figure 1F**). Interestingly, while CD3^-^ and CD8^+^ early apoptotic cells decreased over time, late apoptotic populations increased, suggesting a dynamic progression from early to late apoptosis upon CVF exposure (**Figures 1E, F**). Given that CD3^-^ cells were significantly affected by CVF-induced apoptosis, we further investigated monocyte subpopulations to determine their susceptibility, since monocytes are also the second largest cell population in PBMCs after lymphocytes (35). Monocytes were purified from PBMCs using magnetic-activated cell sorting (MACS) and categorized into classical (CD14^+^CD16^-^) and non-classical (CD14^dim^CD16^+^) monocytes. Our findings confirmed the pro-apoptotic potential of CVF, demonstrating a time-dependent shift from early to late apoptosis in both monocyte subsets (**Figure 1G**). Of note, monocytes were very sensitive to spontaneous cell apoptosis, especially in the first 24 h of culture as reported elsewhere (36, 37).

In summary, our findings demonstrate that degenerating *T. solium* cysts actively drive apoptosis, rather than non-apoptotic death (necrosis), in peripheral immune cell populations. This effect was particularly pronounced in CD8^+^ T cells and monocytes. These results underscore the selective targeting of immune cells by degenerating cyst-derived molecules, further implicating their role in NCC-associated systemic immune inflammation and dysregulation.

### Cyst degeneration drives T and B cell infiltration and microglia/macrophage activation in brains of naturally infected pigs

**Figure 2:**
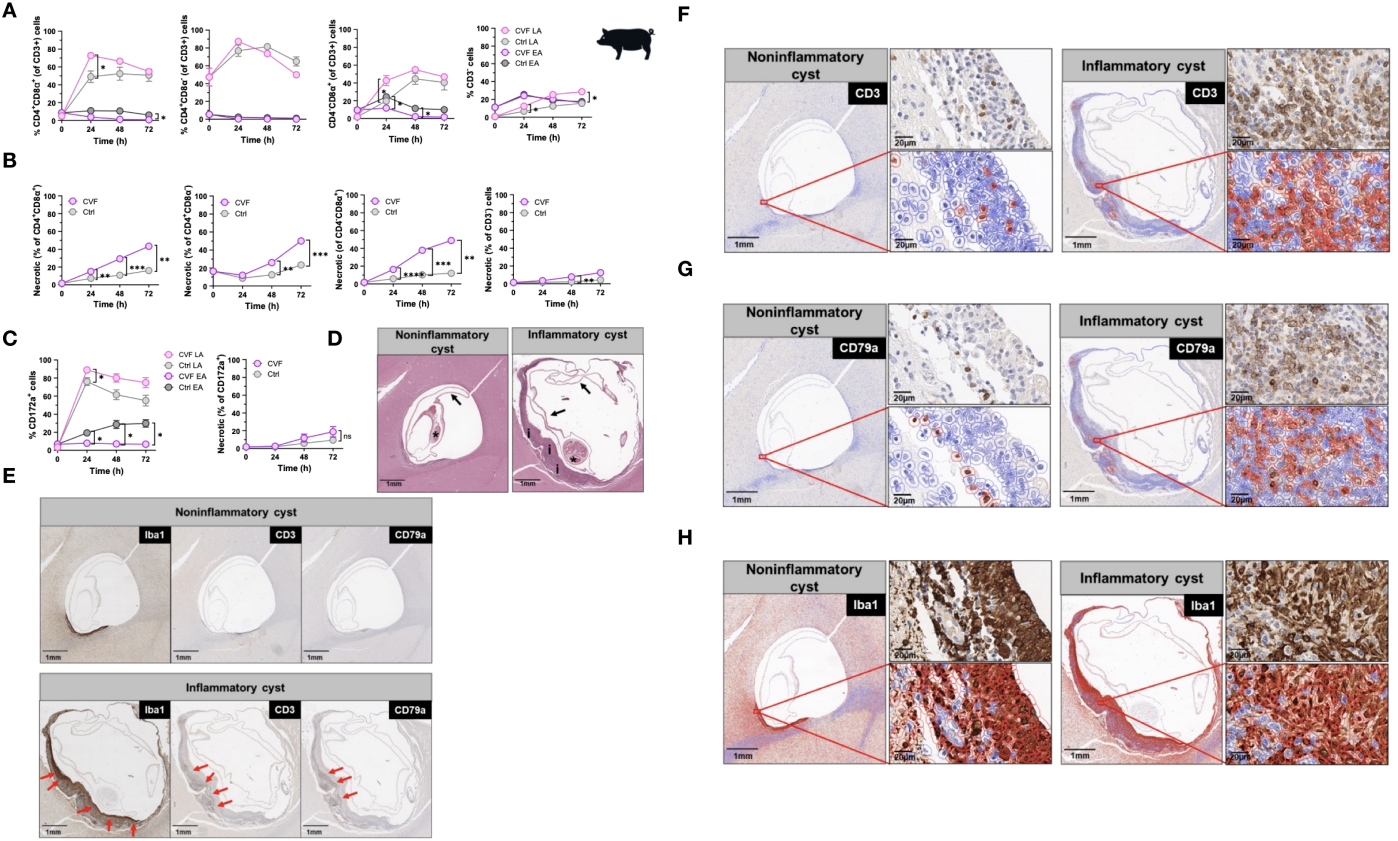
Cyst degeneration drives T and B cell infiltration and microglia/macrophage activation in brains of naturally infected pigs. **(A, B)** Time-dependent induction of early and late apoptosis **(A)** and necrosis **(B)** by CVF in porcine CD4^+^CD8α^+^, CD4^+^CD8α^-^, CD4^-^CD8α^+^, and CD3^-^ cell populations. **(C)** Time-dependent induction of early and late apoptosis (left panel) and necrosis (right panel) in porcine CD172a^+^ monocytes. **(D, H)** Paraffin-embedded naturally infected pig brain slices were stained for characterization of T. solium cyst stage and inflammatory cell infiltrates. **(D)** Staining of viable/noninflammatory (left panel) and degenerating/inflammatory (right panel) cysts according to (42) and (38). Arrows indicate the collapsed bladder wall, the asterisks display the scolex of the parasite, and “i” shows the inflammatory infiltrate around the degenerating cyst. **(E)** Localization of the macrophage/microglia (Iba^+^), T (CD3^+^), B (CD79^+^) cell infiltrates (red arrows) surrounding the cyst in infected pig brain. **(F, H)** Quantification of CD3^+^ T **(F)**, CD79a^+^ B **(G)** cells and Iba1^+^ macrophage/microglia **(H)** (red-bordered cells) in cyst surrounding tissue assessed by QuPath Software. Original image sections shown on upper right panels for each cyst stage. Data information: **(A–C)** Graphs show data from 8 different samples. Statistical analysis was performed using a Two-way ANOVA followed by Tukey’s multiple comparison test. Data are represented as means ± SEM. **(D, H)** Images were analyzed using the Software QuPath [v0.5.1]. *P < 0.05; **P < 0.01; ***P < 0.001; ****P < 0.0001.

Pigs serve as natural hosts for *T. solium* cysts and their infection mirrors in many ways human NCC in terms of immunopathological responses (9, 15, 38). To extend our findings on CVF-induced apoptosis in host immune cells, we examined the effects of CVF on porcine peripheral immune cells and assessed its relevance to the CNS by analyzing immune responses in naturally infected pigs harboring granulomas with intact and degenerating *T. solium* cysts in the brain. Porcine PBMCs were treated with CVF under the same conditions as human PBMCs, and apoptosis was assessed in CD3^+^ T cells, including major subsets (CD4^+^CD8α^-^, CD4^-^CD8α^+^, CD4^+^CD8α^+^), as well as in CD172a^+^ monocytes (**Figures 2A-C**). Unlike humans, pigs possess a unique population of CD4^+^CD8α^+^ double-positive T cells, which are thought to originate from CD4^+^ helper T cells that acquire CD8α expression following antigen recognition (39, 40). Similar to human cells, early apoptosis in porcine PBMCs declined over time, promoting late stages of apoptosis, which peaked at 24 h post-CVF treatment, with a delayed response in CD4^-^CD8α^+^ T cells (**Figure 2A**). However, in contrast to human T lymphocytes, CVF-dependent necrosis increased significantly over time in all porcine lymphocyte populations (**Figure 2B**). In addition, late apoptotic cells steadily increased in the CD3^-^ compartment over time, while CD3^+^ lymphocytes displayed a reduction in late apoptosis after 48 h of treatment (**Figures 2A–B**). Porcine monocyte subsets are primarily identified by CD172a, CD14, and CD163 expression (41). Given that all porcine monocytes express CD172a (41), we focused on CD172a^+^ monocytes for better comparability with human monocytes. Similar to porcine T lymphocytes, CD172a^+^ monocytes showed a peak in late apoptosis at 24 h post-CVF treatment, which declined at later time points (**Figure 2C**). Necrosis also increased over time in monocytes, although this change was not statistically significant (**Figure 2C**). These results suggest that while CVF predominantly induces apoptosis in human immune cells, it promotes a substantial degree of necrosis in porcine immune cells. This indicates a species-specific variation in the mechanisms of degenerated cyst-induced cell death.

In humans, the most severe clinical manifestations of NCC, including recurrent seizures and progressive headaches, are often associated with degenerating cysts encased within granulomatous structures in the brain (8). To investigate the immunopathological relevance of CVF-induced apoptosis *in vivo*, we first analyzed immune cell infiltrates in brain sections from five NCC-infected pigs, comparing regions surrounding degenerating “inflammatory” *T. solium* cysts to those surrounding viable “non-inflammatory” cysts (38, 42). Employing a combination of HE staining with spatial analysis of CD3^+^ T and CD79^+^ B lymphocytes and the Iba1^+^ activated macrophages/microglia, we identified 18 cysts classified into three stages according to the classification used by Gutierrez and Singh (38, 42). In brief, viable cysts (noninflammatory) were classified as cysts with a well-stainable parasite with intact scolex and bladder wall and no edema or inflammation in the surrounding tissue. Degenerating cysts (inflammatory) had an intact bladder wall and scolex, but nuclear disintegration and a mixed inflammatory or granulomatous reaction around the cysts were observed. Although calcified cysts were also detected, no defined structure was found in these cysts. Of the 18 cysts, 1 was a viable cyst (noninflammatory), 11 were degenerating (inflammatory), and 6 were calcified or unclassifiable cysts. Out of the 11 inflammatory cysts, 6 had clearly visible parasites with scolex and are thus included in the present investigation. Representative images of a noninflammatory and an inflammatory cyst from the same pig are shown in **Figure 2D**. Immunohistochemical staining for CD3, CD79a, and Iba1 revealed a distinctive localization of immune cells within granulomatous structures. Iba1^+^ activated macrophages/microglia were predominantly located on the inner side of the granuloma, adjacent to degenerating cysts (**Figures 2E, H**). In contrast, CD3^+^ T cells and CD79a^+^ B cells were primarily found in the outer layers of the granuloma (**Figures 2E, G**). Quantitative analysis indicated an approximately three-fold increase in CD3^+^ T cell infiltration (**Figures 2E, F**) and a ten-fold increase in CD79a^+^ B cell infiltration (**Figures 2E, G**) around inflammatory cysts compared to non-inflammatory cysts (**Figures 2E, G**; **Supplementary Figures S3A, B**).

These findings provide compelling evidence that cyst-derived vesicular fluid products contribute to peripheral immune cell apoptosis and inflammation. Moreover, cyst degeneration is associated with significant immune cell infiltration in the CNS, highlighting its potential role in NCC immunopathogenesis.

### CVF-induced cell apoptosis is modulated preferentially through the caspase 9 pathway

**Figure 3:**
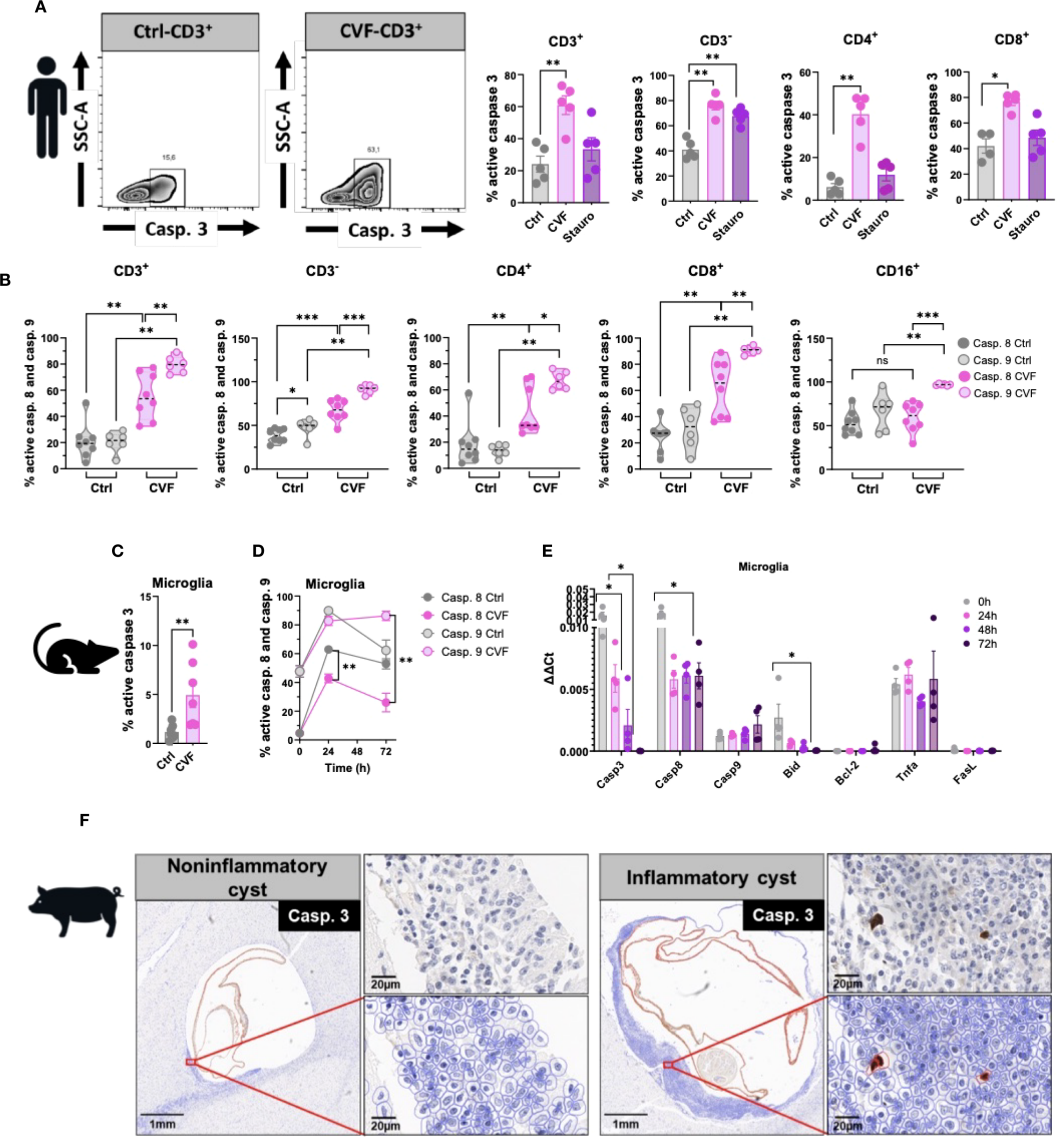
CVF-induced cell apoptosis is modulated preferentially through the caspase 9 pathway. **(A)** Active caspase 3 induction in CD3^+^ and CD3^-^ and in CD4^+^ and CD8^+^ cell populations by 72h stimulation of human PBMC by CVF as compared to the positive control staurosporine. **(B)** Activity of cleaved caspases 8 and 9 in CD3^+^, CD3^-^, CD4^+^, CD8^+^ T cells and CD16^+^ monocytes as detected by FLICA assays following 72h stimulation with CVF. **(C)** Active caspase 3 induction in murine microglia at 72h. **(D)** FACS analysis of time-dependent modulation of cleaved caspase 8 and 9 activity in murine microglia by CVF. **(E)** Time-dependent expression of apoptosis related genes in microglia modulated by CVF. **(F)** Naturally infected pig brain slices stained for caspase 3-expressing cells surrounding inflammatory and non-inflammatory cysts assessed by QuPath Software. Original image sections shown on upper right panels for each cyst stage. Data information: Graphs show data from 4–8 different samples. Statistical analysis was performed using a Mann-Whitney test **(A–C)**, a Two-way ANOVA followed by Tukey’s multiple comparison test **(D, E)**. Data are represented as means ± SEM. Images were analyzed using the Software QuPath [v0.5.1]. *P < 0.05; **P < 0.01; ***P < 0.001.

Several signaling pathways contribute to apoptosis, with the extrinsic pathway initiated by death receptors, and the intrinsic pathway that occurs through the mitochondria, being the main ones (43). To determine which apoptotic pathways are activated by CVF, we assessed caspase activity, given that caspase 3 serves as a key effector in both apoptotic mechanism pathways (44). CVF treatment led to a significant increase in CD3^+^ T cell-expressing active caspase 3, including both CD4^+^ and CD8^+^ subsets, compared to untreated and staurosporine-treated positive controls (**Figure 3A**). To distinguish between the extrinsic and intrinsic apoptosis pathways, we evaluated the activation of initiator caspases for both pathways: caspase 8 (extrinsic) and caspase 9 (intrinsic) (44). Using fluorochrome-labeled inhibitor of caspase (FLICA) assays, we observed significant activation of both caspase 8 and caspase 9 in CD3^+^, CD4^+^, and CD8^+^ T cells, as well as in CD3^-^ non-T cells (**Figure 3B**). However, in monocytes, only caspase 9 was activated, with no detectable caspase 8 activity (**Figure 3B**). Of note, CVF treatment shifted the entire monocyte compartment toward CD16^+^-expressing monocytes. Despite the involvement of both pathways in lymphocytes, caspase 9 activation was significantly higher than caspase 8, suggesting a predominant role of the intrinsic mitochondrial pathway in CVF-induced apoptosis. An analogous pattern was found in microglia, where CVF-induced caspase 3 activation (**Figure 3C**) correlated with a progressive increase in cleaved caspase 9 levels, whereas cleaved caspase 8 decreased sharply over time (**Figure 3D**).

To determine whether CVF stimulation modulated gene expression of caspases, we performed qRT-PCR analysis (**Figure 3E**). Caspase 9 expression increased over time, although not statistically significant, while caspase 3 and caspase 8 expression declined. Additionally, we analyzed key mediators influencing caspase 8 and caspase 9 activation. TNFα and FasL, two primary inducers of the extrinsic apoptotic pathway (23), showed no significant changes in mRNA levels. Similarly, the expression of Bcl-2, an anti-apoptotic protein (45), remained unchanged. In contrast, Bid exhibited a marked decline. Of note, Bid is a pro-apoptotic protein and active inducer of mitochondrial outer membrane permeabilization that bridges caspase 8 activation to the extrinsic pathway (45). This suggests that the caspase 8 - Bid axis is not a major contributor to CVF-induced apoptosis. To explore the relevance of these findings *in vivo*, we stained brain tissue sections from NCC-infected pigs for caspase 3. Apoptotic caspase 3^+^ cells were detected exclusively in areas surrounding degenerating inflammatory cysts (**Figure 3F**).

Taken together, these findings indicate that CVF predominantly activates the mitochondrial apoptosis pathway via caspase 9, leading to caspase 3 activation and immune cell death. Furthermore, the presence of apoptotic cells in the CNS near degenerating cysts underscores the potential role of CVF-induced apoptosis in NCC immunopathogenesis.

### CVF treatment causes loss of mitochondrial membrane potential and dysfunction

**Figure 4:**
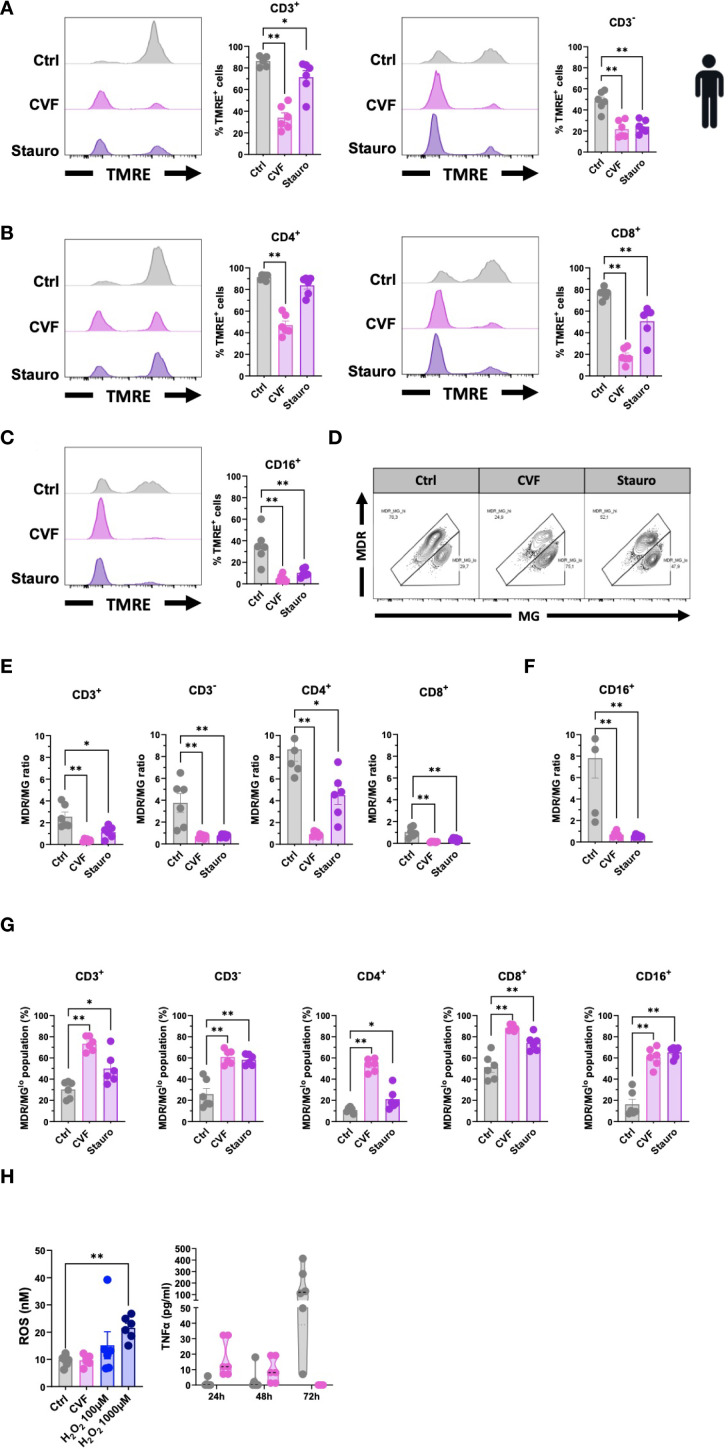
CVF treatment causes loss of mitochondrial membrane potential and dysfunction. **(A–C)** Assessment of mitochondrial damage and dysfunctionality in CD3^+^ and CD3^-^ T cells **(A)**, CD4^+^ and CD8^+^ T cells **(B)**, and CD16^+^ monocytes **(C)** following 72h CVF treatment of human PBMC. **(D–F)** Evaluation of the mitochondrial activity per mass (MDR/MG ratio) in T cells and monocytes. **(D)** FACS plots characterizing MDR/MG ratio in CD3^+^ T cells. **(E, F)** MDR/MG ratio in CD3^+^, CD3^-^, CD4^+^, and CD8^+^ T cells **(E)**, and in CD16^+^ monocytes **(F)**. **(G)** MDR/MG^lo^ cell populations with increased mitochondrial mass but reduced transmembrane potential following CVF stimulation in T cell populations and monocytes. **(H)** Evaluation of intracellular ROS activity in human PBMC following 72h stimulation with CVF (left panel). H_2_O_2_ is used as a positive control. Culture supernatants were collected at different time points, and levels of TNFα (right panel) were analyzed. Data information: Graphs show data from 6 different samples. Statistical analysis was performed using a Mann-Whitney test and a Kruskal-Wallis test followed by a Dunn’s multiple comparison test. Data are represented as means ± SEM. *P < 0.05; **P < 0.01.

Our data demonstrate that *T. solium* CVF preferentially activates the intrinsic apoptosis pathway to promote cell death. A hallmark of this pathway is mitochondrial dysfunction, characterized by the release of cytochrome C from the mitochondria and a loss of mitochondrial transmembrane potential (44, 46). We thus next assessed the mitochondrial integrity following CVF exposure using the TMRE and MitoTracker assays in human PBMC. As depicted in **Figure 4A**, CVF-treated cells exhibited a significant reduction in TMRE fluorescence and signal, indicating mitochondrial transmembrane potential loss and depolarization across CD3^+^ T cell and CD3^-^ non-T cell compartments, as well as CD4^+^ and CD8^+^ T cells (**Figure 4B**), and CD16^+^ monocytes (**Figure 4C**). MitoTrackers are cationic fluorescent probes that accumulate inside mitochondria due to the high mitochondrial transmembrane potential and are useful tools to visualize mitochondrial mass, integrity, and morphology (47). We used MitoTracker Deep Red (MDR) and MitoTracker Green (MG) to assess mitochondrial membrane potential and mitochondrial mass, respectively (34). The MDR/MG ratio is considered a parameter for mitochondrial activity per mass (34). As **Figures 4D, F** show, we observed a marked decrease in the MDR/MG ratio, suggesting an impairment of mitochondrial activity relative to mass for the lymphocyte (**Figures 4D, E**) and CD16^+^ monocyte (**Figure 4F**) populations treated with CVF and Staurosporine as compared to the untreated controls. This was accompanied by an increase in MDR/MG^lo^ populations (**Figure 4G**), further supporting mitochondrial dysfunction.

Mitochondrial impairment involves many upstream mediators and cellular stress signals in the induction of the intrinsic apoptosis pathway such as mitochondrial DNA damage, heat shock, hypoxia, ER stress, and reactive oxygen species (ROS) (23, 45, 48–51). We tested the involvement of intracellular ROS activity using a fluorometric ROS assay in human PBMC as compared to TNFα, a marker of the extrinsic pathway (**Figure 4H**), and to provide further mechanistic insights. We detected no difference in intracellular ROS levels between the CVF-treated and control cells, while TNFα decreased over time in CVF-stimulated PBMCs. Thus, treatment with CVF led to cellular mitochondrial damage and depolarization, associated with impaired mitochondrial function.

### Symptom development in NCC patients increases cellular apoptosis sensitivity and soluble FasL mediator in the periphery

**Figure 5:**
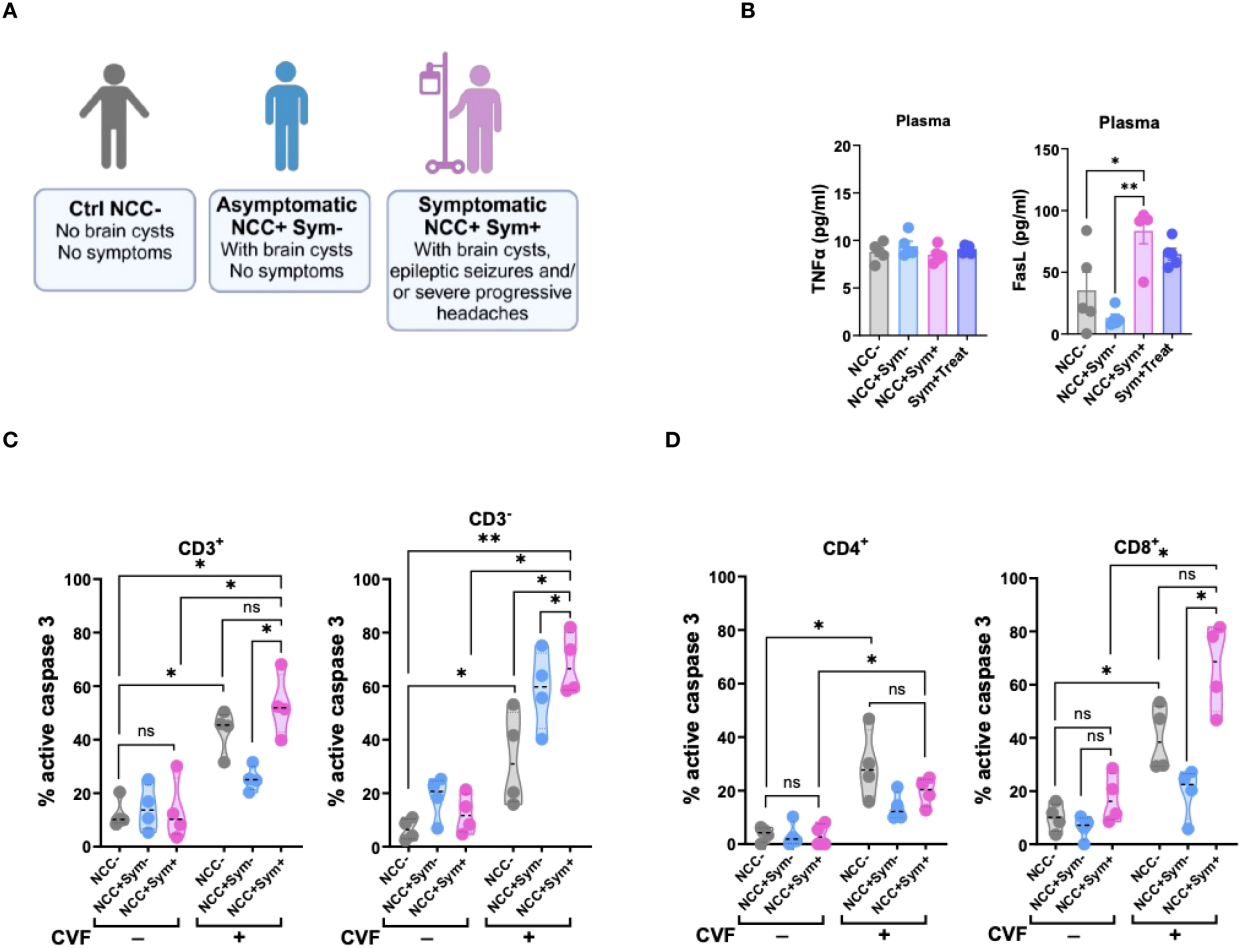
Symptomatic NCC patients show elevated signs of T cell apoptosis and soluble FasL mediator in the periphery. **(A)** Illustration of NCC patient groups and controls. **(B)** Evaluation of TNFα (left panel) and FasL (right panel) levels in the plasma of NCC patients and controls. **(C, D)** Caspase 3 activity in CD3^+^ and CD3^-^ **(C)**, and in CD4^+^ and CD8^+^ **(D)** T cell populations following 72h CVF stimulation of PBMC from uninfected (NCC-) and infected asymptomatic (NCC^+^Sym^-^) and symptomatic (NCC^+^Sym^+^) NCC patients as compared to unstimulated controls. Data information: Graphs show data from 4–6 different samples. Patients were age-matched. Statistical analysis was performed using a Mann-Whitney test and Kruskal–Wallis one‐way ANOVA followed by a Dunn’s multiple comparison test **(B–D)**. Data are represented as means ± SEM. *P < 0.05; **P < 0.01.

To investigate whether markers for apoptosis such as FasL or TNFα would be detectable in symptomatic NCC patients, we made use of samples from our previous study (28). In this study, we had recruited a cohort of Tanzanian participants, including healthy controls and NCC-infected asymptomatic and symptomatic patients harboring active lesions with degenerating cysts in the brain (28) (**Figure 5A**). To assess the involvement of apoptosis-associated soluble mediators in NCC, we measured plasma levels of TNFα and FasL in the different patient groups (**Figure 5B**). TNFα levels did not significantly differ across the cohort groups. However, symptomatic NCC patients exhibited significantly higher plasma levels of FasL compared to asymptomatic patients and healthy controls (**Figure 5B**). Notably, asymptomatic patients had a trend of lower FasL levels than healthy controls. These findings suggested that degenerating cysts may contribute to the elevated levels of the pro-apoptotic mediator FasL.

To further explore the relevance of caspase 3-driven apoptosis in symptomatic NCC, we assessed caspase 3 activity in PBMCs from patients after stimulation with CVF *ex vivo* (**Figures 5C, D**). In the absence of stimulation, caspase 3 activity was similar between the different groups. However, upon CVF exposure, PBMCs from symptomatic NCC patients exhibited a marked increase in caspase 3 activity across all cell populations, except CD4^+^ T cells, when compared to healthy controls. In contrast, PBMCs from asymptomatic NCC patients demonstrated a relatively weaker response to CVF, except for CD3^-^ cells, when compared to the controls (**Figures 5C, D**).

Taken together, these results indicate a strong association between elevated levels of the soluble inflammatory mediator FasL and caspase-driven apoptosis with symptomatic disease in NCC.

### Early and late stages of apoptosis are not induced by cysteine proteases but distinct factors enriched in CVF

**Figure 6:**
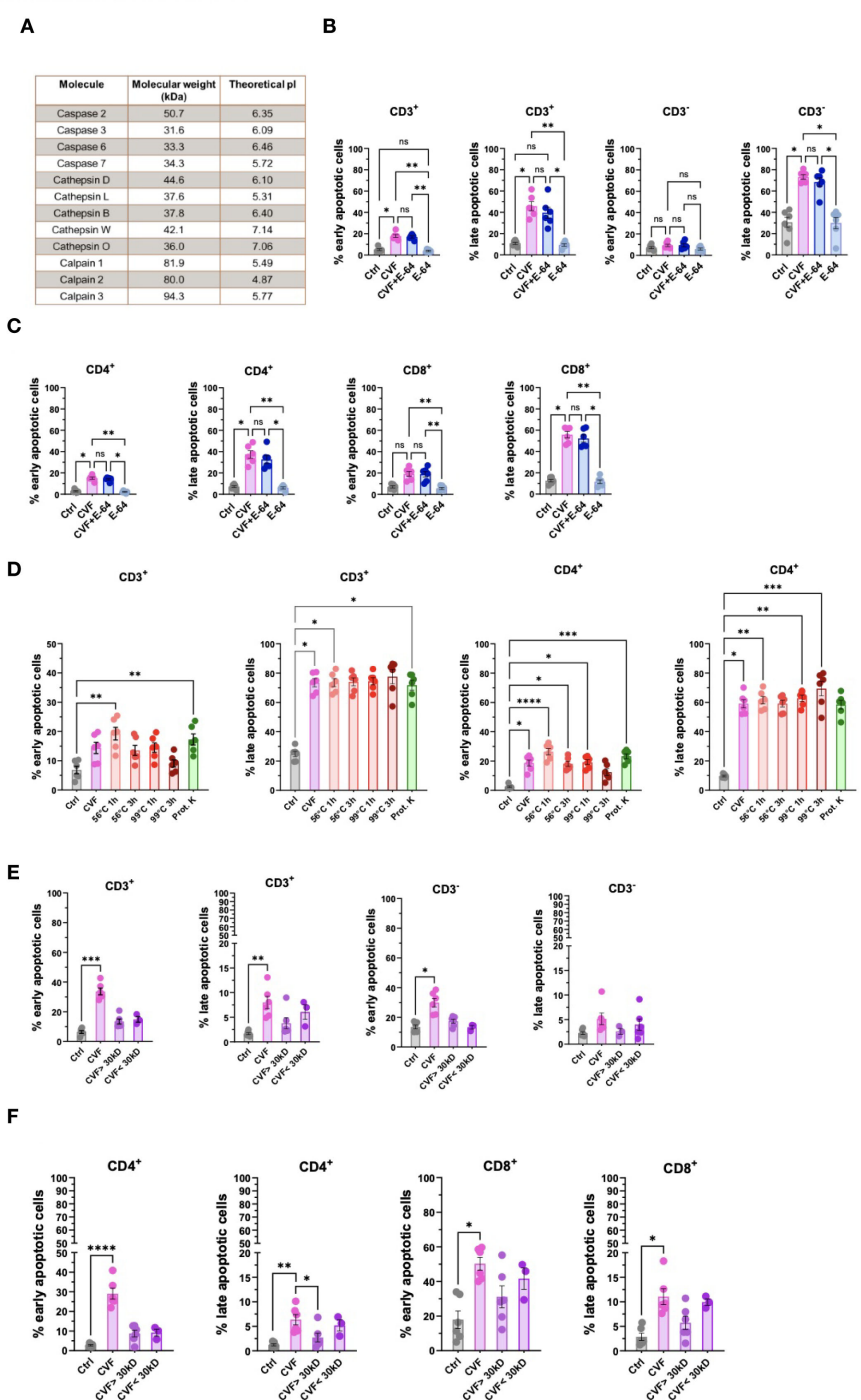
Early and late stages of apoptosis are not induced by cysteine proteases but distinct factors enriched in the CVF. **(A)** Identification of cysteine proteases and apoptosis signaling pathway-related molecules present in CVF by differential mass spectrometry. **(B, C)** Effect of cysteine protease inhibitor E-64 on CVF-induced early and late apoptosis in CD3^+^ and CD3^-^ **(B)**, and in CD4^+^ and CD8^+^ **(C)** T cells in PBMC after 72h. **(D)** Effect of heat treatment and proteinase K digestion of CVF on the induction of early and late apoptosis stages in CD3^+^ and CD4^+^ T cells. **(E, F)** Column fractionation of CVF and induction of early and late apoptosis stages in CD3^+^ and CD3^-^ **(E)** and CD4^+^ and CD8^+^ **(F)** T cell populations. Data information: The mass spectrometry data was analyzed using WormBase Parasite database for *T. solium* (Version WS282, October 2022). Graphs show data from 6 different samples. Statistical analysis was performed using Kruskal-Wallis test followed by a Dunn’s multiple comparison test. Data are represented as means ± SEM. *P < 0.05; **P < 0.01; ***P < 0.001; ****P < 0.0001. ns, non-significant.

Having demonstrated that *T. solium* CVF induced apoptosis preferentially through the caspase 9-mediated mitochondrial pathway in various immune cell populations, we next sought to identify specific CVF molecules responsible for this pro-apoptotic effect. To achieve this, we conducted a differential mass spectrometry analysis of CVF components. Among the identified CVF molecules (3), we detected 12 proteins, namely cysteine proteases, known to be associated with apoptotic pathways, including the caspases (2, 3, 6, and 7), cathepsins (B, D, L, O, and W), and calpains (1, 2, and 3) (52, 53) (**Figure 6A**). To determine the role of cysteine proteases in CVF-induced apoptosis, we pretreated PBMCs with E-64, an irreversible cysteine protease inhibitor (54), and assessed apoptosis levels. Surprisingly, E-64 pretreatment did not prevent CVF-induced early or late apoptosis in CD3^+^ and CD3^-^ cells (**Figure 6B**), nor in CD4^+^ and CD8^+^ T cells (**Figure 6C**), indicating that cysteine proteases may not be essential mediators of CVF-driven apoptosis.

We next evaluated the biochemical properties of the active pro-apoptotic molecules in CVF by subjecting CVF to heat treatment (56 °C and 99 °C), as well as proteinase K digestion, to assess the involvement of protein-based factors (**Figure 6D**; **Supplementary Figure S4**). While heat treatment at 99 °C for 3 hours mildly reduced early apoptosis induction in CD3^+^, CD4^+^ (**Figure 6D**), and CD8^+^ T cells (**Supplementary Figure S4**), overall, treatment at lower temperature and with proteinase K did not significantly alter CVF-induced apoptosis. Accordingly, cysteine proteases and heat- or proteinase K-sensitive proteins did not appear to be the primary drivers of CVF-mediated late-stage apoptosis.

Next, we investigated the molecular size of the active pro-apoptotic factors. We fractionated CVF into molecules smaller or larger than 30 kDa and assessed the pro-apoptotic potency. We found that molecular size with the chosen cut-off did not significantly impact the apoptotic potency of CVF. However, we observed a trend to stronger pro-apoptotic effects, mostly late apoptosis, by molecules < 30 kDa, as compared to molecules > 30 kDa in CD3^+^, CD3^-^ cells (**Figure 6E**), and CD4^+^ and CD8^+^ T cells (**Figure 6F**).

**Figure 7:**
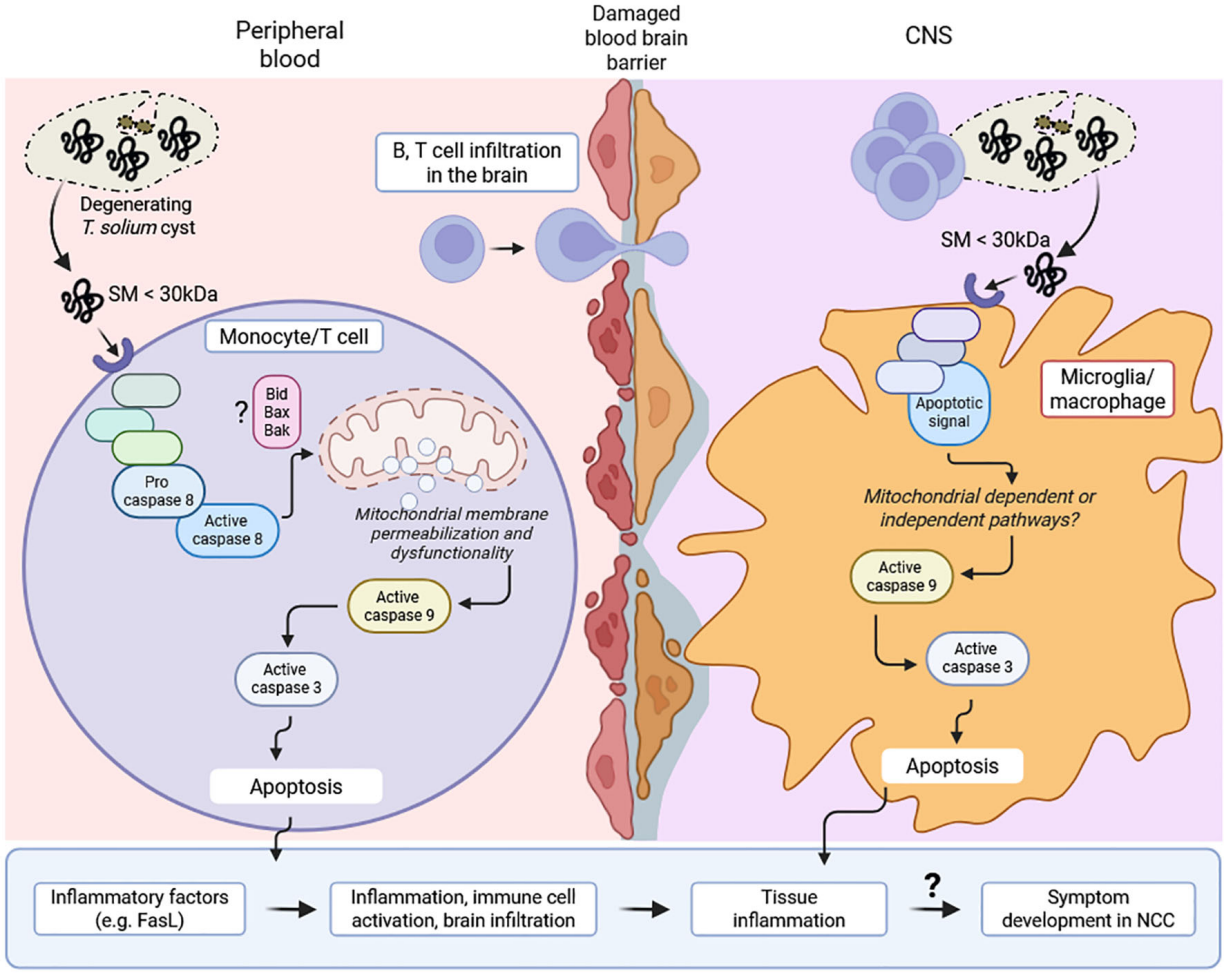
Proposed model of CVF-induced apoptosis mechanisms and implications during NCC. Cyst degeneration releases small molecules (SM) (< 30 kDa), which induce apoptotic signal within peripheral cells (monocyte, T cell) and microglia/macrophage in the central nervous system (CNS). This signal activates caspase 8 cascade and mitochondrial membrane permeabilization leading to caspase 9 accumulation. This initiates caspase 3-associated apoptosis, immune cell activation and inflammatory factors such as FasL. Similarly, CNS inflammation following mitochondrial-dependent or independent-caspase 9-induced apoptosis in microglia/macrophage drives immune cell (B, T cell) infiltration in the brain. The increasing cell death and ongoing inflammation may eventually drive a disruption in the structure and function of neuronal synapse and excitability, leading to the development and progression of symptoms (e.g. epilepsy) in NCC patients.

These results indicated that CVF-mediated apoptosis was driven by small or a combination of small molecular weight components rather than heat- and proteinase-sensitive proteins. The identification of these pro-apoptotic molecules may provide insights into immune-mediated inflammation and disease exacerbation during NCC. A proposed model of CVF-induced apoptosis mechanisms and implications during NCC is presented in **Figure 7**.

## Discussion

This study highlights the pivotal role of caspase 9-mediated apoptosis in *T. solium* CVF-induced cell death at peripheral and central nervous system levels, underscoring its relevance in NCC-associated inflammation. This pathway, potentially initiated by small molecules rather than proteins or enzymes, drives significant loss of mitochondrial membrane potential, increased mitochondrial mass and dysfunction, ultimately leading to cellular apoptosis. Clinically, CVF, used in this study as a surrogate to mimic degenerating cysts, promoted caspase activation associated with higher serum levels of pro-apoptotic FasL during symptom development in patients with NCC. These findings, supported by the detection of caspase 3^+^ cells around degenerating cysts in the brain of NCC-infected pigs, highlight a link between parasite-driven apoptosis, disease pathogenesis, and severity.

Previous investigations have demonstrated the apoptosis of CD3^+^ lymphocytes in the inflammatory infiltrate around *T. solium* cysts in infected pig brains (19, 55), with a potential role of caspase 3 (55). Further studies revealed that the cysts release Annexin B1, which binds to eosinophils and neutrophils and induces apoptosis through the induction of Ca^2+^ influx. Interestingly, Annexin B1 had no proapoptotic effect on lymphocytes (56). Our discovery of the intrinsic apoptotic pathway activation involving caspase 9 is particularly important in the context of neuroinflammatory diseases, where mitochondrial dysfunction and programmed cell death contribute to disease pathology and progression (20, 44, 50). Our data support the idea that mitochondrial integrity is severely compromised upon CVF exposure, leading to a significant loss of mitochondrial transmembrane potential across multiple immune cell subsets, including CD4^+^ and CD8^+^ T cells and monocytes. Importantly, these findings reinforce the central role of mitochondrial damage in CVF-induced immune cell death, aligning with previous reports on helminth-derived molecules (e.g. *S. mansoni*-derived apoptosis-inducing factor, *B. pahangi* antigen (BpA), excretory/secretory-products of *E. multilocularis* larvae), interfering with host cell viability, activation, and metabolism (57–62). Our data reveal a notable species-specific difference in the type of cell death induced by *T. solium* CVF, with porcine immune cells exhibiting a higher degree of necrosis compared to predominantly apoptosis in human cells. Several factors could explain this divergence. First, pigs possess a distinct composition of peripheral immune cell subsets, including a high frequency of double-positive (CD4^+^CD8^+^) T cells, absent in humans, which may have differential sensitivity to parasitic antigens or stress-induced cell death pathways (63, 64). Second, interspecies differences in the expression or density of pattern recognition receptors (e.g., TLRs, CLRs), cytokine receptors, and death receptors (e.g., Fas, TNFR) could alter cellular susceptibility to CVF components. Third, metabolic profiles and redox homeostasis differ between human and pig and may affect mitochondrial integrity and cellular stress responses, thereby influencing the threshold for apoptosis versus necrosis (65). Moreover, species-specific differences in protease expression and stability (e.g., cathepsins) may also modulate downstream death pathways. Importantly, naturally infected pigs often harbor cysts at various developmental stages, and the presence of both viable and degenerating cysts in the brain reflects a chronic infection state. This chronicity could influence the cellular microenvironment, including immune cell composition, activation status, and susceptibility to apoptosis, as compared to acute infection. For example, chronic cyst fluid antigenic stimulation may prime resident immune cells such as microglia and infiltrating monocytes toward a more apoptosis-susceptible phenotype, particularly via mitochondrial stress and caspase 9 activation, as observed in our data. These findings highlight the complexity of modeling human NCC in animal systems when translating immune responses across species. Future comparative studies employing transcriptomic or receptor profiling approaches may provide better mechanistic insight into these interspecies variations and help refining porcine models for human-relevant translational research and neurological diseases.

Inflammatory conditions such as multiple sclerosis, Alzheimer’s disease, and other neurodegenerative disorders have been linked to activation of caspase 9 and mitochondrial apoptosis pathways (27, 66). Given that mitochondrial damage leads to the release of Cytochrome c and subsequent caspase 9 activation, our data thus suggests furthermore that the mitochondrial pathway may be crucial in immune-mediated neuroinflammation. Notably, following CVF exposure, we observed a progressive increase of caspase 9 activity in microglia, which reside in granulomas around degenerating cysts. We have previously shown that CVF abrogates phagocytic function in microglia, resulting in the release of the pro-epileptic mediator TGF-β (3). In this study, we show now that this may result from prolonged apoptosis. Importantly, the loss of microglia in inflammatory infiltrates through apoptosis and the reduced phagocytosis of dying cells and dead cell debris due to the CVF impacting microglia, may potentially contribute to epileptogenesis. Indeed, effective microglial phagocytosis of apoptotic cells and degenerated neuronal remnants is essential for minimizing CNS inflammation and related symptoms by preserving healthy neuronal networks (24, 67).

Epileptic seizures are the most common symptom of NCC, with 70-90% of symptomatic NCC patients being affected (6). The mechanisms driving epileptic manifestations in NCC are, to date, not fully understood. In line with our findings, the conditional knockout of apoptosis-signaling-kinase 1 (ASK-1) in microglia led to decreased seizure severity and histological damage in a kainic acid-induced mouse seizure model (68). ASK-1 is activated under stress conditions, driving the activation of the mitochondrial apoptosis pathway through phosphorylation of c-Jun N-terminal kinase (JNK) and p38 MAPK (23, 69). Furthermore, in a mouse model of NCC, doxycycline treatment-dependent inhibition of mitochondrial apoptosis and subsequent Cytochrome c release (70) resulted in reduced mortality and diminished levels of neuronal apoptosis and inflammatory infiltrates in the CNS (71). CNS inflammation driven by the pro-apoptotic effect of CVF may not only contribute to epileptogenesis, but further instigate the breakdown of the blood-brain barrier (72, 73). As a consequence, increased exposure of the brain to inflammatory soluble mediators from the periphery, such as serum albumin, has been shown to induce inflammatory TGF-β-signaling in astrocytes, leading to excitatory synaptogenesis and reduced glutamate uptake capacity (72, 73).

A key aspect of our study is the identification of peripheral host soluble mediators associated with apoptosis in NCC patients. Symptomatic NCC patients exhibited significantly elevated FasL levels in the plasma and increased caspase 3-dependent apoptosis susceptibility of peripheral blood cells, thus potentially also contributing to NCC symptom development. Binding of FasL to Fas receptor (CD95) results in the recruitment of the Fas-associated death domain (FADD), leading to autoactivation of procaspases 8 and 10, causing the activation of the effector caspases 3 and 7 (23, 74). In line with our findings, Chen et al. found significantly elevated FasL levels in the cerebrospinal fluid of NCC patients, with NCC patients with calcified active lesions in the brain displaying higher serum levels of FasL levels compared to uninfected controls (75). Indeed, elevated FasL levels have been found in various neuroinflammatory conditions (76, 77). This could have important implications for understanding disease severity (e.g. neurological symptom development) and therapeutic interventions in symptomatic NCC patients. Nonetheless, the observed discrepancy between the dominant caspase 9-mediated intrinsic apoptosis pathway *in vitro* and the elevated levels of the extrinsic pathway mediator FasL detected in the plasma of symptomatic NCC patients likely reflects the interconnected, dynamic and complex nature of the regulation of apoptotic mechanisms *in vivo*. Indeed, following cyst degeneration and CVF leaking, it is plausible that FasL can be upregulated by activated T cells, macrophages, and microglia in response to chronic antigenic stimulation, proinflammatory cytokines (such as TNFα, IFNγ), or cell stress, all of which are features of active NCC lesions (4, 10, 30). Furthermore, crosstalk between the extrinsic and intrinsic pathways is well-established. FasL-induced caspase 8 activation can amplify mitochondrial dysfunction via Bid cleavage and truncation, while caspase 9-mediated intrinsic apoptosis may, in turn, promote FasL expression through feedback signaling (45, 78). Therefore, FasL elevation in symptomatic patients may not be the primary trigger of apoptosis but rather reflect a downstream consequence of ongoing tissue inflammation and immune activation. Thus, the clinical setting introduces additional inflammatory factors that can upregulate FasL and engage the extrinsic apoptosis pathway. Furthermore, different immune cell populations may exhibit varying apoptotic sensitivities, with some relying more on Fas-FasL interactions during chronic infection. Also, the lower FasL levels in asymptomatic patients could reflect a reduced engagement of Fas–FasL–mediated apoptotic pathways in the peripheral circulation of individuals harboring latent infection. This may contribute to the maintenance of immune homeostasis and protection from overt inflammatory responses, which aligns with the clinically silent status of these patients associated with immunosuppression and higher Treg levels (3, 4, 14, 79). In addition, in our study, we did not detect statistically significant differences in TNF-α levels between symptomatic, asymptomatic NCC patients, and uninfected controls. Indeed, TNF-α is a highly dynamic cytokine with a short half-life in circulation, and its levels are influenced by the timing of sample collection relative to disease progression and stimulating antigen load in circulation. It is possible that in our cross-sectional sampling design, peak TNF-α levels had already declined or fluctuated. Moreover, TNF-α exerts much of its pathological function in NCC locally within the CNS, rather than in the periphery (18, 30, 80, 81). Thus, systemic levels may not fully reflect the localized inflammatory milieu within the CNS. While TNF-α plays a role in NCC-associated inflammation, our data suggest that peripheral TNF-α levels alone may not serve as a reliable surrogate marker of disease severity or for apoptotic activity. Increasing the group sample size would provide further biological relevance for this immune aspect. This suggests that apoptosis in NCC is context-dependent, with the intrinsic pathway being potentially more relevant during early parasite-host interactions, while the extrinsic pathway may become prominent as neuroinflammation progresses with distinct cyst stages and interacting molecules.

To elucidate the molecular determinants of CVF-mediated apoptosis, we employed mass spectrometry analysis and functional inhibition assays. While CVF contains cysteine proteases such as caspases, cathepsins, and calpains, their inhibition via E-64 did not prevent apoptosis, indicating that alternative non-protease factors contribute to the observed effects or suggesting that the pro-apoptotic activity is not solely dependent on classical cysteine protease activity. This contrasts with previous reports, suspecting cysteine proteases being the cause of the proapoptotic effect of *T. solium* metacestode excretory-secretory (E/S) products (82, 83). These differences may be explained by the use in our setting of CVF products and not E/S products, which may contain different levels and groups of cysteine proteases. Furthermore, our biochemical fractionation experiments revealed that the active pro-apoptotic molecules in CVF are likely to be small (< 30 kDa) and resistant to proteinase K digestion. Annexin B1 from *T. solium* metacestodes has been postulated to have proapoptotic effects on innate immune cells (e.g. eosinophils) activating the intrinsic apoptosis pathway through induction of calcium influx (56). Although we identified annexin B2 from our mass spectrometry analysis, its molecular weight (MW) is > 30 kDa. Also, tetraspanins, protease inhibitors, and other non-identified small peptides in the hydatid cyst fluid released from *E. granulosus* can interfere with host immune signaling, contributing to apoptosis in certain immune and cancer cells (84, 85). This raises intriguing possibilities regarding parasite-derived metabolites or other small molecules that could be modulating host cell apoptosis. Notably, several of the identified pro-apoptotic molecules in CVF, including cathepsin L, cathepsin S, and caspases, have known functional subunit fragments ranging between 10–30 kDa (86, 87). It remains plausible that small active forms or degradation products of these proteases contribute to apoptosis through non-canonical mechanisms, or that other bioactive metabolites, peptides, or post-translationally modified fragments below 30 kDa may be involved. The heat- and protease-resistant nature of the apoptogenic activity suggests a role for non-pure protein or highly stable small molecules, possibly of metabolic origin (88). Thus, non-protein molecules, including lipids, small peptides, and potentially parasite-derived extracellular vesicles (EVs) containing miRNAs (e.g., miR-71, miR-146) may modulate host signaling pathways related to apoptosis and mitochondrial function (89, 90). However, conventional mass spectrometry may not fully resolve these candidates, especially if they are low-abundance, chemically modified, or highly hydrophobic. Future investigations integrating untargeted metabolomics, peptidomics, and high-resolution small molecule profiling, potentially through LC-MS/MS, NMR, or ultrasound probe and size and activity-guided fractionation, are needed to definitively identify the active components. Given the established role of mitochondrial apoptosis in neuroinflammatory diseases, future studies should aim to characterize these CVF-derived apoptogenic molecules and their potential as therapeutic targets.

In conclusion, our study provides novel mechanistic insights into how *T. solium* cyst fluid molecules manipulate host cell apoptosis, particularly through caspase 9 activation, and its role in mitochondrial dysfunction and immune-driven neuroinflammation in NCC. Our findings contribute to a better understanding of NCC progression and pathogenesis. This offers avenues to develop strategies for identification of symptomatic NCC patients via peripheral biomarkers such as FasL, and to mitigate parasite-induced immune dysregulation to eventually improve clinical outcomes in NCC patients.

## Data Availability

The original contributions presented in the study are included in the article/**Supplementary Material**. Further inquiries can be directed to the corresponding author.
