# Supplementary material for: Helminthic larval stage induces cellular apoptosis via caspase 9-mediated mitochondrial dysfunction
Source: Front Immunol. 2025 Sep 25;16:1603385. doi: 10.3389/fimmu.2025.1603385 (PMC12507911; doi:10.3389/fimmu.2025.1603385)
Supplement: Supplementary file 1 [file DataSheet1.pdf]

Supplementary Material

Supplementary Figure S1:

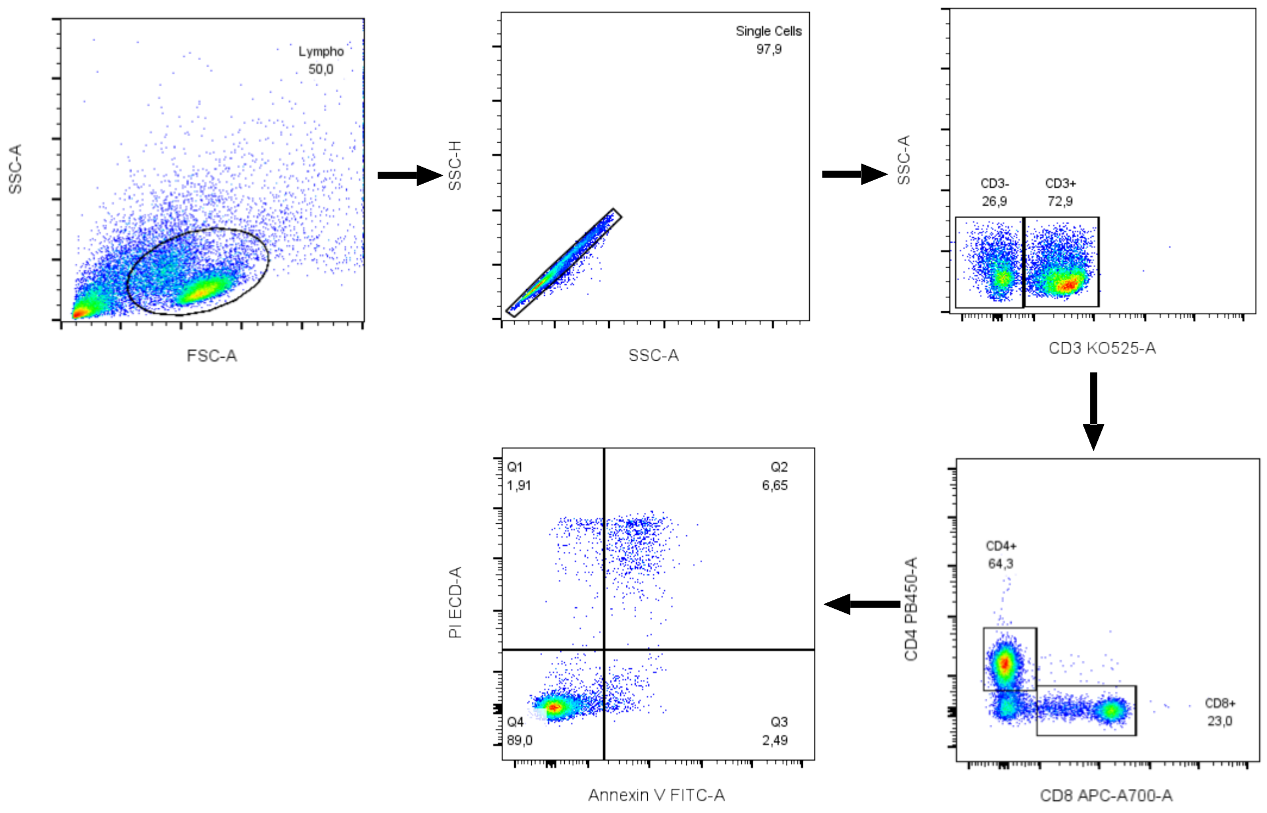

Supplementary Figure S1: FACS gating strategy for early (Annexin V+/PI-) and late (Annexin V+/PI+) apoptotic as well as necrotic (Annexin V-/PI+) cell populations.

Supplementary Figure S2:

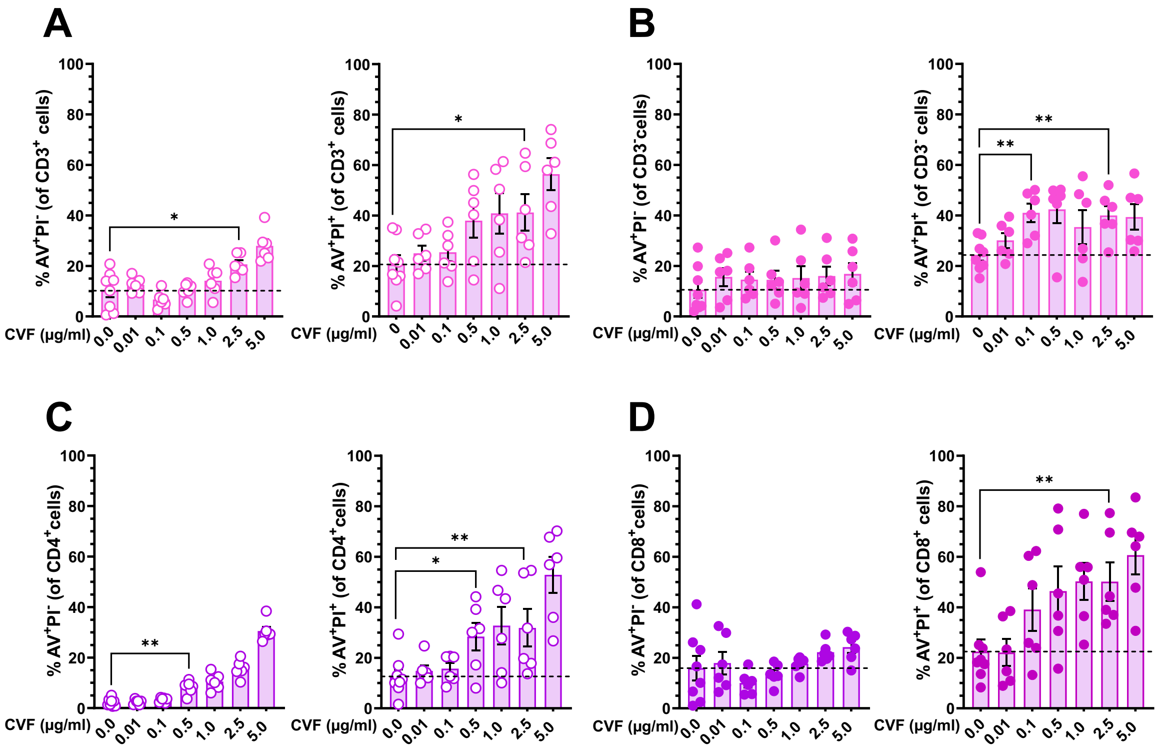

Supplementary Figure S2: CVF concentration-dependent induction of early (Annexin V^+/PI^-) and late apoptosis (Annexin V^+/PI^+) in $CD3^+$ (A), $CD3^-$ (B), $CD4^+$ (C) and $CD8^+$ (D) T cells in human PBMC after 72h.

Data information: Graphs show data from 6-8 different samples. Statistical analysis was performed using a Mann Whitney test and a Kruskal-Wallis test followed by a Dunn‘s multiple comparison test. Data are represented as means ± SEM. $^*P < 0.05$; $^{**}P < 0.01$

Supplementary Figure S3:

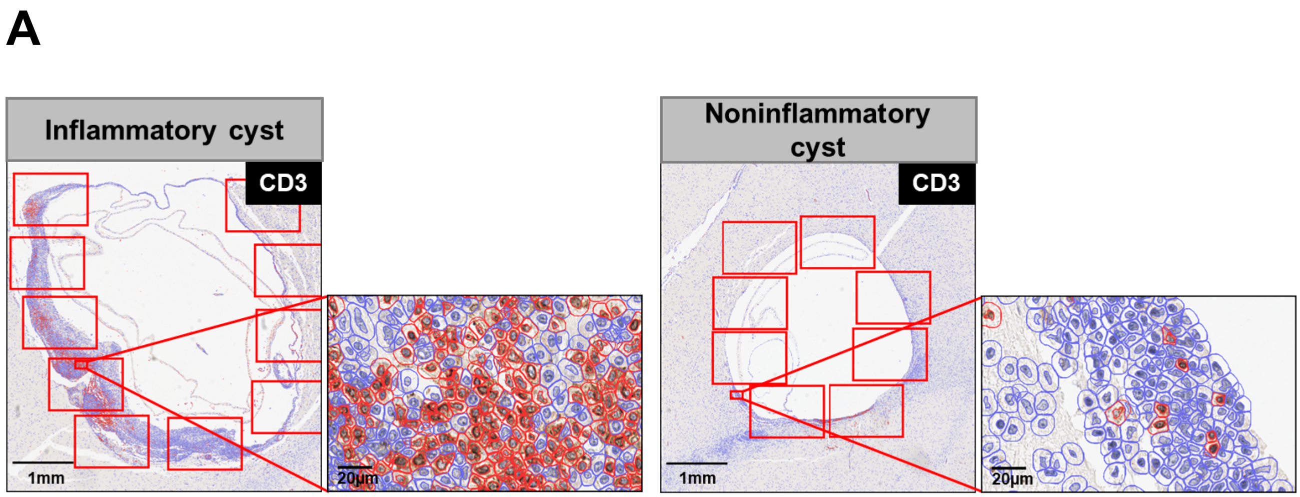

CD3					
**Inflammatory (degenerating) cyst**			**Noninflammatory (viable) cyst**		
	Num Pos	Positive %		Num Pos	Positive %
**Rectangle 1**	556	22.7	**Rectangle 11**	1	0.17
**Rectangle 2**	287	11.1	**Rectangle 12**	21	4.2
**Rectangle 3**	819	20.7	**Rectangle 13**	15	4.0
**Rectangle 4**	1751	27.4	**Rectangle 14**	132	7.8
**Rectangle 5**	829	22.5	**Rectangle 15**	213	22.5
**Rectangle 6**	252	4.4	**Rectangle 16**	6	1.0
**Rectangle 7**	100	9.8	**Rectangle 17**	5	0.9
**Rectangle 8**	198	19.6	**Rectangle 18**	7	1.1
**Rectangle 9**	89	8.3			
**Rectangle 10**	41	3.7			
**Mean**	492.2	15.0	Mean	50	5.2

B

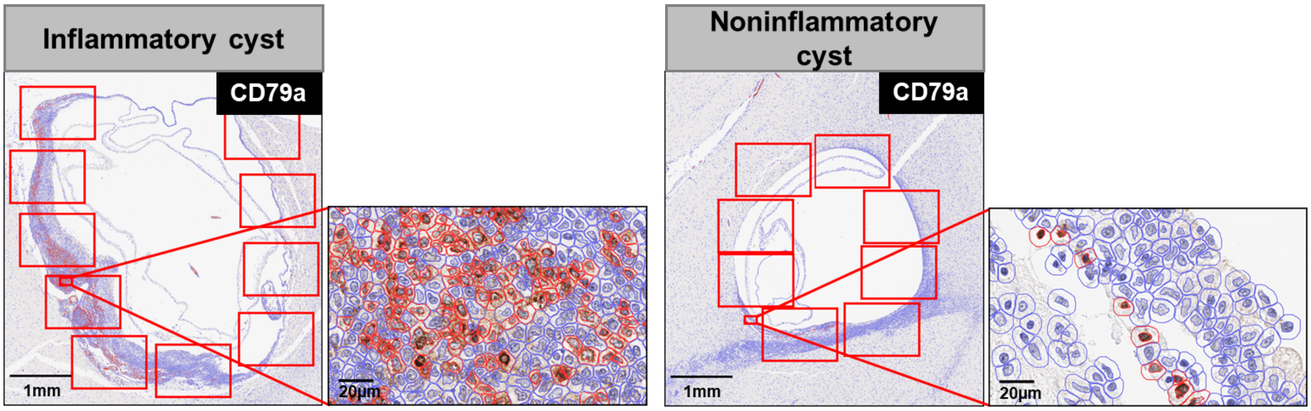

CD79a					
**Inflammatory (degenerating) cyst**			**Noninflammatory (viable) cyst**		
	Num Pos	Positive %		Num Pos	Positive %
**Rectangle 1**	326	12.4	**Rectangle 11**	5	0.7
**Rectangle 2**	334	12.0	**Rectangle 12**	13	1.5
**Rectangle 3**	752	16.7	**Rectangle 13**	16	2.0
**Rectangle 4**	1453	22.4	**Rectangle 14**	104	2.5
**Rectangle 5**	591	13.2	**Rectangle 15**	3	0.1
**Rectangle 6**	143	2.5	**Rectangle 16**	0	0
**Rectangle 7**	9	0.8	**Rectangle 17**	2	0.2
**Rectangle 8**	30	2.8	**Rectangle 18**	2	0.1
**Rectangle 9**	15	1.6			
**Rectangle 10**	14	1.4			
**Mean**	366.7	8.6	**Mean**	18.1	0.9

Supplementary Figure S3: Quantification of $CD3^{+}$ (A) and $CD79a^{+}$ (B) cells in cyst surrounding tissue. 8-10 rectangles (1,25mm x 0,875mm) for automated cell counting were drawn around one

representative of each inflammatory and non-inflammatory cyst, and positive cells were counted automatically with QuPath [v0.5.1].

Supplementary Figure S4:

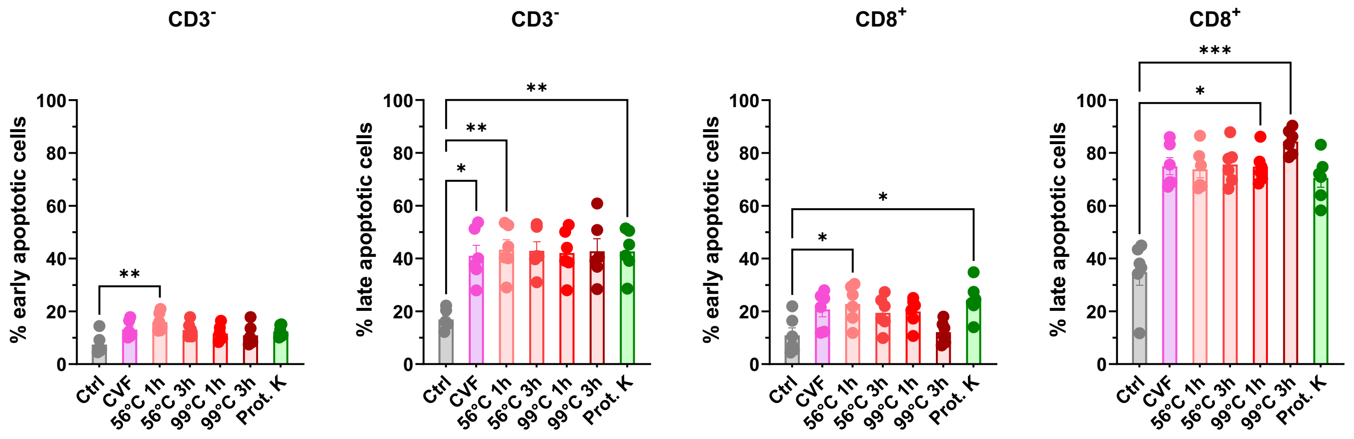

Supplementary Figure S4: Effect of heat treatment and proteinase K digestion of CVF on the induction of early and late apoptosis stages in $CD3^-$ and $CD8^+$ T cells.

Data information: Graphs show data from 6 different samples. Statistical analysis was performed using Kruskal-Wallis test followed by a Dunn‘s multiple comparison test. Data are represented as means ± SEM. *P < 0.05; **P < 0.01; ***P < 0.001
